# Supplementary material for: Combining mass spectrometry and machine learning to discover bioactive peptides
Source: Nat Commun. 2022 Oct 20;13:6235. doi: 10.1038/s41467-022-34031-z (PMC9584923; doi:10.1038/s41467-022-34031-z)
Supplement: Supplementary file 1 — Supplementary Information [file 41467_2022_34031_MOESM1_ESM.pdf]

Supplementary Information

Combining mass spectrometry and machine learning to discover bioactive peptides

Christian T. Madsen*, Jan C. Refsgaard, Felix G. Teufel, Sonny K. Kjærulff, Zhe Wang, Guangjun Meng, Carsten Jessen, Petteri Heljo, Qunfeng Jiang, Xin Zhao, Bo Wu, Xueping Zhou, Yang Tang, Jacob F. Jeppesen, Christian D. Kelstrup, Stephen T. Buckley, Søren Tullin, Jan Nygaard-Jensen, Xiaoli Chen, Fang Zhang, Jesper V. Olsen, Dan Han, Mads Grønborg, Ulrik de Lichtenberg.

This file includes:

Supplementary Figures 1-17 and legends
Supplementary Note 1
Supplementary References

Uniprot	Protein	N-term.	Peptide sequence	C-term.	Peptide name	Enzymes	Organelle	Observed	Ref.
P55897	Histone H2A	- M	AGRGKQGGKVRAKAKTRSSRAGLQFPVGRVHRLLRKGNY	QR	Buforin I	Pepsin Ca / Cb	Extracellular	(Partially)	1
P11859	Angiotensinogen	AG	DRVYIHPF	HL	Angiotensin II	ACE	Extracellular	No	2
O08677	Kininogen-1	AR	RPPGFSPFR	SV	Bradykinin	Kallikrein (plasma)	Extracellular	No	3
P22005	Proenkephalin-A	KK	YGGFM	KR	Met-enkephalin	Cathepsin L + Arg/Lys-aminopeptidase	Secretory pathway	No†	4
P62329	Thymosin-β4	- M	Ac-SDKP	DM	Hematopoietic reg. peptide	Meprin-α + prolyl-oligopeptidase	Extracellular	No†	5
P01946	Hemoglobin α	VD	PVNFKFLSH	CL	Hemopressin	Endopeptidase 24.15 + neurolysin	Cytosol	Yes	6
P01946	Hemoglobin α	VL	TSKYR	- -	Neokyotorphin	Cathepsin D + aspartyl protease	Unknown	No†	7
P02088	Hemoglobin β-1	LL	VVYPWTQRY	FD	VV-hemorphin-7	Cathepsin D + aspartyl protease	Unknown	Yes	8
Q92686	Neurogranin	DD	PGANAAAAKIQASFR	GH	FE2	Proteasome + Thimet-oligopeptidase	Cytosol	Yes	9
P23435	Cerebellin-1	RS	GSAKVAFSAIRSTNH	EP	[des-Ser1]-cerebellin (FE4)	Proteasome + Thimet-oligopeptidase	Cytosol	Yes	9
P12074	Cytochrome C, 6A1	PM	SSGAHGEEGSARMWKT	LT	FE3	Proteasome + Thimet-oligopeptidase	Cytosol	Yes	9
P01270	Parathyroid hormone	KR	SVSEIQLMHNLGKHLNSMERVEWLRKKLQDVHNF	VA	1-34	Cathepsin D	Endosome	No§	10
Q9NRV9	Heme-binding protein 1	- -	Ac -MLGMIKNSLFGSVETWPWQVL	SK	F2L	Cathepsin D	Unknown	No	11
P53621	Coatomer alpha	- -	MLTKFETKSARVKGLSFHPKRPWIL	TS	Xenin-25	Cathepsin E	ER	No	12

Supplementary Figure 1: Bioactive peptides formed through non-canonical processing. Non-canonical bioactive peptides with flanking amino acids. Proposed enzymatic activities and likely localization of peptide formation is indicated. Observation status designates whether the peptide was found in the mice tissues studied here (liver, muscle, ileum, brain, pancreas, epididymal fat and subcutaneous fat). (†) the peptide is below 7 amino acids in length rendering it undetectable by the search conditions used here. (§) the TrEMBL entry in mouse (Q9Z0L6) awaits manual annotation and therefore is undetectable with the search conditions used here. Ac, Acetylation.

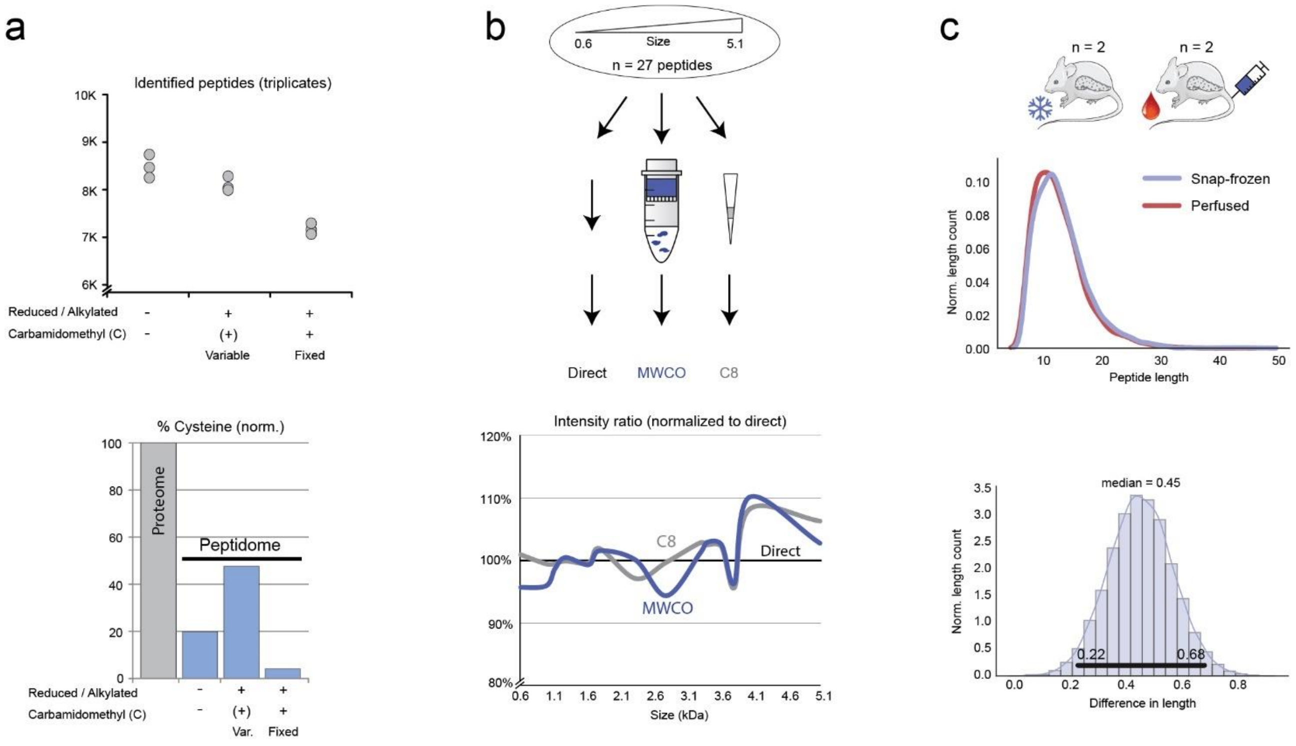

Supplementary Figure 2: Experimental setup.

a, Triplicates of pancreatic tissue samples were reduced/alkylated and compared to untreated samples. The reduced-alkylated samples were searched with carbamidomethyl as either variable or fixed modification on cysteine residues. The *Mus musculus* proteome (ID: UP000000589) with its baseline number of relative cysteine residues set at 100%. A small but significant drop in identification rate was observed when reducing/alkylating. Source data are provided as a Source Data file. **b**, Experiment to rule out potential bias induced by MWCO. A pool of n=27 peptides between 0.6 – 5.1 kDa were split in three and 1 µg from each group injected either directly, through a 10 kDa molecular weight cut-off (MWCO) filter, or purified over a C8 stagetip. Intensities normalized to the direct injection. **c**, Kernel density plot of peptide length distribution. Anaesthetized animals were perfused with a saline solution containing protease inhibitors[13]. The pancreas was removed and snap frozen and compared to pancreas samples where the perfusion step was omitted. Both perfused and non-perfused samples subsequently undergo heat inactivation in a Denator T1 stabilizor. Density plot of mean peptide length based on 10.000 draws with replacements with median and 2.5% and 97.5% quantiles (lower panel). Mouse and tissue images were produced using Servier Medical Art (https://smart.servier.com/).

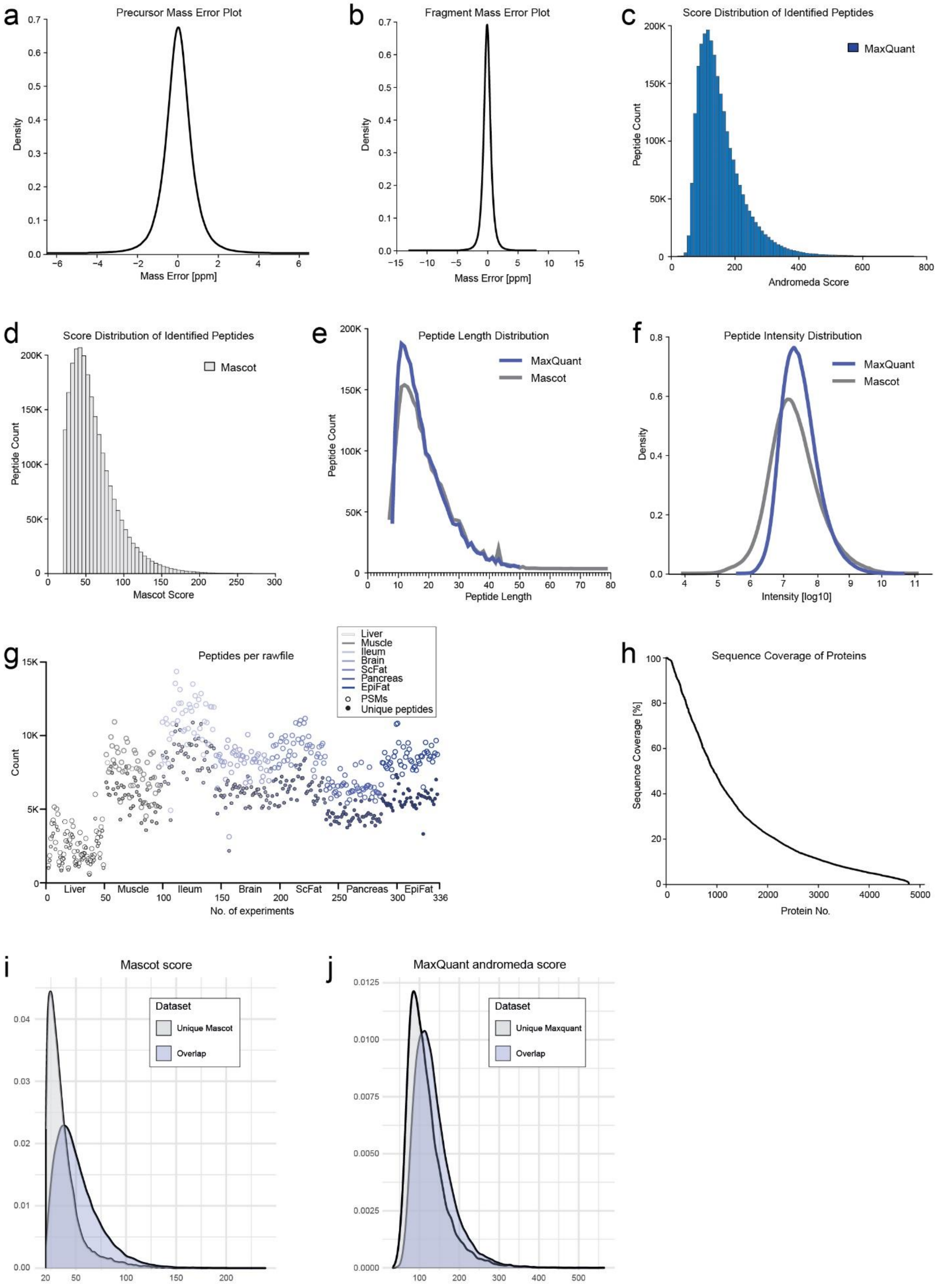

Supplementary Figure 3: See next page for caption.

Supplementary Figure 3: Global MS data quality.

a, Precursor mass error in parts-per-million (ppm) density plot for all identifications observed in the MaxQuant dataset. **b**, Fragment ion mass error in ppm density plot for all identifications observed in the MaxQuant dataset. **c**, Andromeda score distribution for all identified peptides by MaxQuant search engine. **d**, Mascot score distribution of all peptides identified with a score above 20. **e**, Peptide length distribution of all identified peptides by MaxQuant and Mascot. Peptides below 7 amino acids were excluded before search in MaxQuant and discarded after search in Mascot. **f**, Density estimations of peptide intensities identified by MaxQuant or Mascot. **g**, Scatter plot of peptide spectrum matches (PSM) and unique peptides identified per rawfile for all seven tissues. **h**, Protein sequence coverage in percentage observed in the MaxQuant dataset. Sorted by decreasing sequence coverage. **i**, Density plot of Mascot scores from uniquely identified peptides and the identified peptides in the overlap of both search engines. **j**, Density plot of andromeda scores from uniquely identified peptides and the identified peptides in the overlap from both search engines.

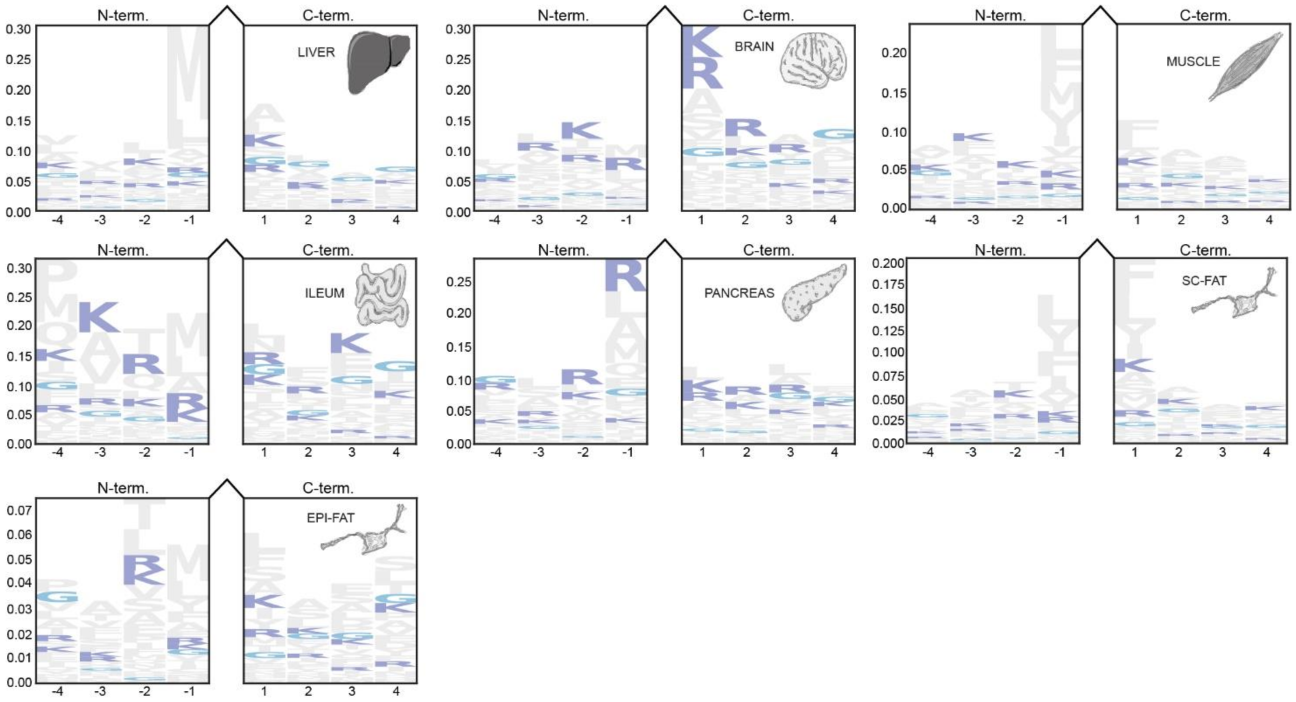

Supplementary Figure 4: Tissue specific flanking motifs.
N- and C-terminal flanking logo plots depicting the Kullback-Leibler divergence between a given tissue, and all other tissues. The divergence is based on a PFM matrix containing the normalized peptide intensity as recorded by the mass spectrometer. Highlighting the deviation across a tissues flanking positions compared to the remaining tissues investigated illustrates how peptide processing preference is unique in each tissue. Tissue images produced using Servier Medical Art (https://smart.servier.com/).

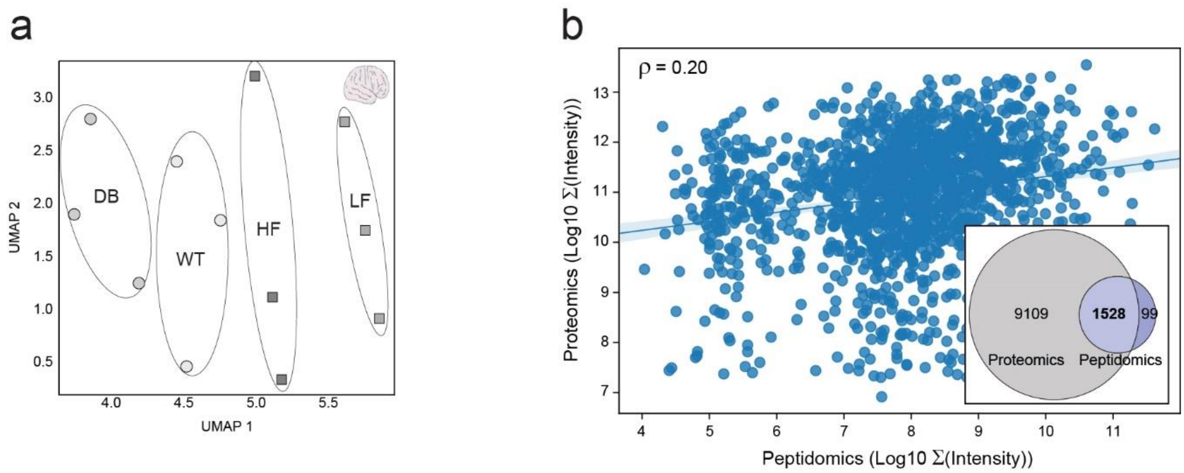

Supplementary Figure 5: Comparison protein and peptide level expression in brain.

a, Conventional shotgun proteomics employing High-pH reverse-phase fractionation was used to quantify >10,000 proteins in the brain. UMAP on top100 most variant proteins consisting of WT, DB, LF, HF mice (n=3 in each group). The strains separate at the level of protein expression similar to what was observed on the peptide level. **b**, Summed intensity compared at the protein level from tryptic peptides (proteomics) and non-tryptic peptides (peptidomics) in n=1528 overlapping proteins in brain. The Pearson correlation coefficient (p=0.20) was calculated from n=3 proteomics samples to any combinatorial average from n=3 peptidomics samples (taken from n=12). The translucent band is the 95% confidence interval. The weak correlation demonstrates that the peptide patterns observed do not simply reflect degradation products of highly abundant proteins. Source data are provided as a Source Data file.

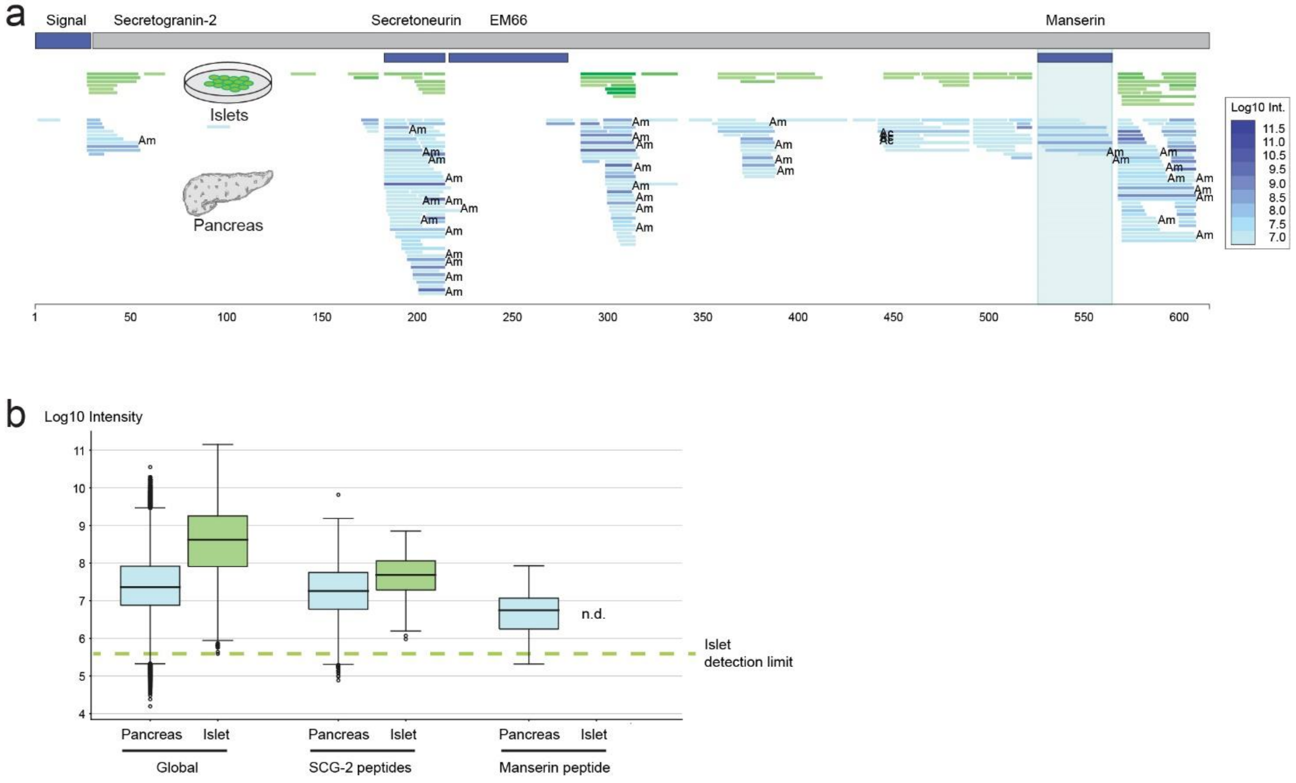

Supplementary Figure 6: Intracellularly stored peptides are detected in whole-tissue peptidomics. **a**, Secretogranin-2 (Uniprot ID: Q03517) and peptides identified in islet (green) and pancreas (blue) with intensity of color indicating peptide abundance (see label). n=4 independent biological replica of 500 islets were stimulated for 1 hour with 500 µM isobutylmethylxanthine and 10 µM forskolin and the media collected. Ac, Acetylation. Am, Amidation. The Manserin peptide (position 527-566) is highlighted to illustrate that this particular peptide is clearly visible in whole-tissue peptidomics but not detected in the islet supernatant. **b**, Log_{10} intensity boxplot of all peptides identified in pancreas samples (n=48 biological independent animals) and islet samples (n=4 independent experiments of 500 stimulated islet cells). The box plot centre is the median, the bounds are the lower and upper quartile values (25th percentile and 75th percentile respectively). The lower whisker extends to the lowest observed value greater than the lower quartile minus 5 times the interquartile range (IQR) of the data, the upper whisker to the highest observed value lower than the upper quartile plus 5 times the IQR. The minimum, lower quartile, median, upper quartile, and maximum are as follows; Pancreas-Global (5.3, 6.9, 7.4, 7.9, 9.5), Islet-Global (5.9, 7.9, 8.6, 9.3, 11.2), Pancreas-SCG-2 (5.3, 6.8, 7.3, 7.8, 9.2), Islet-SCG-2 (6.2, 7.3, 7.7, 8.1, 8.9) and Pancreas-Manserin (5.3, 6.3, 6.7, 7.1, 7.9). The global peptide abundance is compared to all peptides originating from the Secretogranin-2 backbone, as well as peptide intensities originating from Manserin. The lowest abundant peptide detected in islets is a peptide from Transgelin-3 with a log_{10} intensity of 5.85, approximate at 10 times lower intensity than the Manserin peptide in pancreas, and approximate 100 times lower than the average expression level of all other Secretogranin-2 peptides in islets. Source data are provided as a Source Data file.

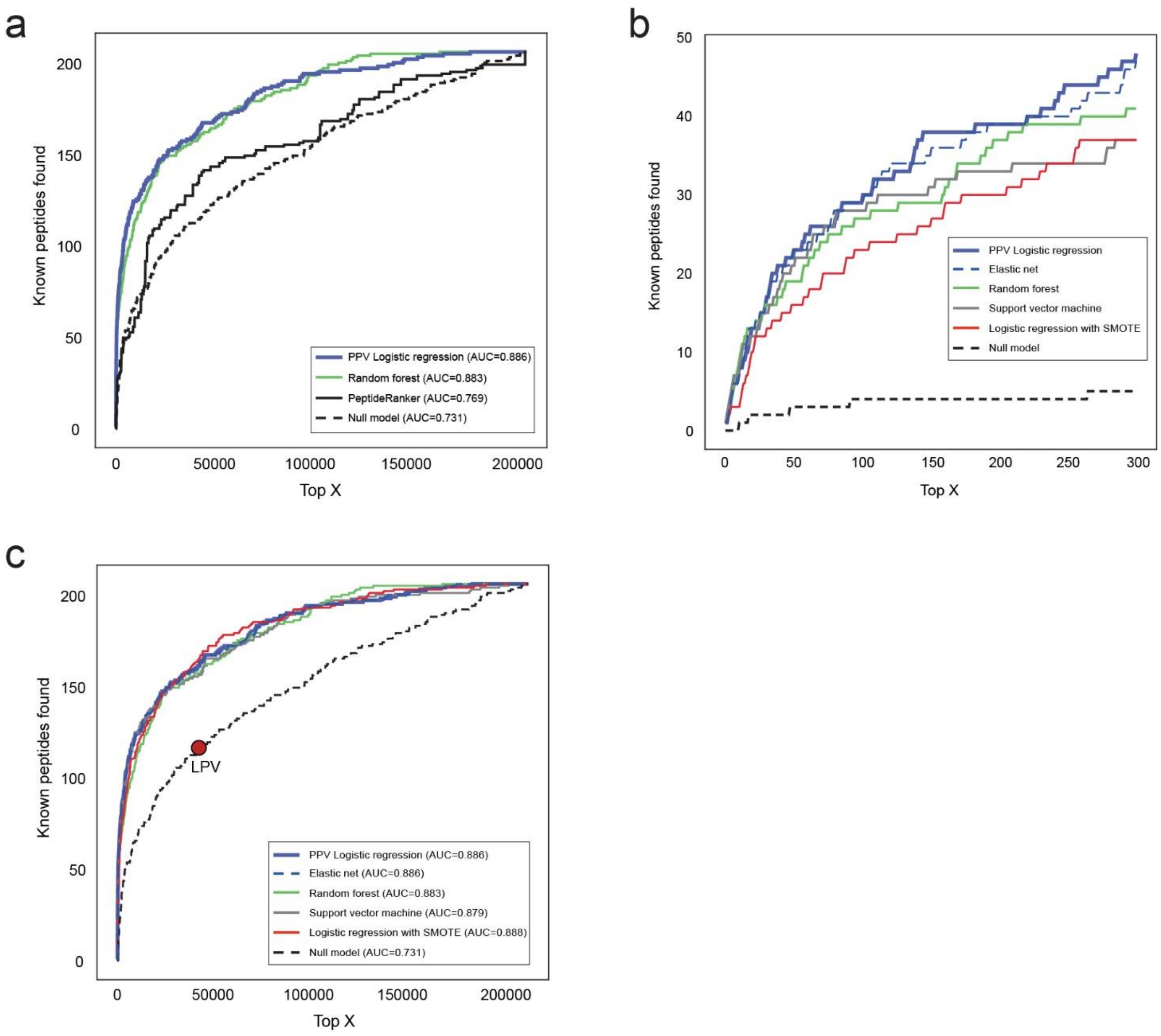

Supplementary Figure 7: Comparing performance of different machine learning models.
a, Number of known annotated peptides and Area-Under-the-Curve (AUC) found by each type of model as a function of rank based scoring, using nested cross-validation in total dataset. PPV model based on Logistic Regression (solid blue), Random forest (green), PeptideRanker (solid black), Null model (dotted black; model uses only a single input feature, the $\log_{10}$ (total peptide abundance). **b**, Number of known annotated peptides found by each type of model in top300 highest scoring peptides. PPV model (Logistic Regression, solid blue), Elastic net (dotted blue), Random forest (green), Support vector machine (solid grey), Logistic regression with SMOTE (solid red), Null model (dotted black). Non-linear models (Random Forest and SVMs) did not confer any advantage over their simpler linear counterparts (Bayesian and simple logistic regression, and no performance benefits were observed with elastic net regularization or SMOTE. **c**, Same models as in b), but in the entire dataset. Longest-Peptide-Variant (LPV) position (red dot; TP=122, FP=52810) determined from (Ref: 13).

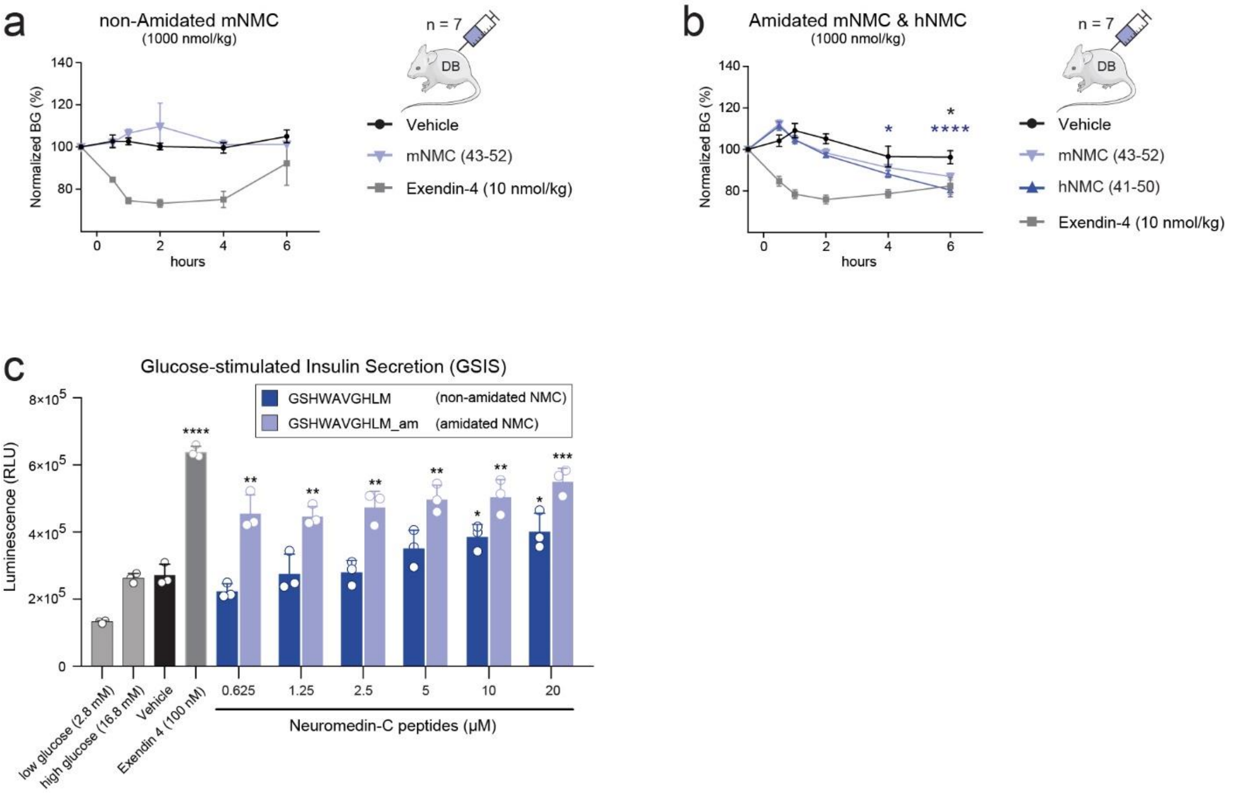

Supplementary Figure 8: Neuromedin-C (Q8R1I2) activity depends on C-terminal amidation.
a, BG mean values ± SEM in n=7 *db/db* mice after non-amidated mNMC peptide (43-52) intraperitoneal injection at 1000 nmol/kg. No effect on BG is observed. **b**, BG mean values ± SEM in n=7 *db/db* mice after amidated mNMC peptide (43-52) and amidated hNMC peptide (41-50) intraperitoneal injection at 1000 nmol/kg. Two-way ANOVA with post-hoc Dunnett's test, $P = 0.0325$ (4h, hNMC), $P = 0.0171$ (6h, mNMC), $P < 0.0001$ (6h, hNMC). **c**, Glucose-stimulated insulin secretion as determined by Relative Luciferase Units ± SD in INS-1E luciferase cells (70,000 cells/well) in n=3 independent measurements. Cells were incubated with non-amidated or amidated neuromedin-C peptide at indicated concentrations or Exendin-4 at 100 nM for 30 min before luminescence measurement. Unpaired two-sided t-test with the following P-values in relation to vehicle control; Exendin-4 ($P < 0.0001$), 0.625_am ($P = 0.0081$), 1.25_am ($P = 0.0025$), 2.5_am ($P = 0.0040$), 5_am ($P = 0.0019$), 10 ($P = 0.0178$), 10_am ($P = 0.0028$), 20 ($P = 0.0261$), 20_am ($P = 0.0008$). Source data are provided as a Source Data file.

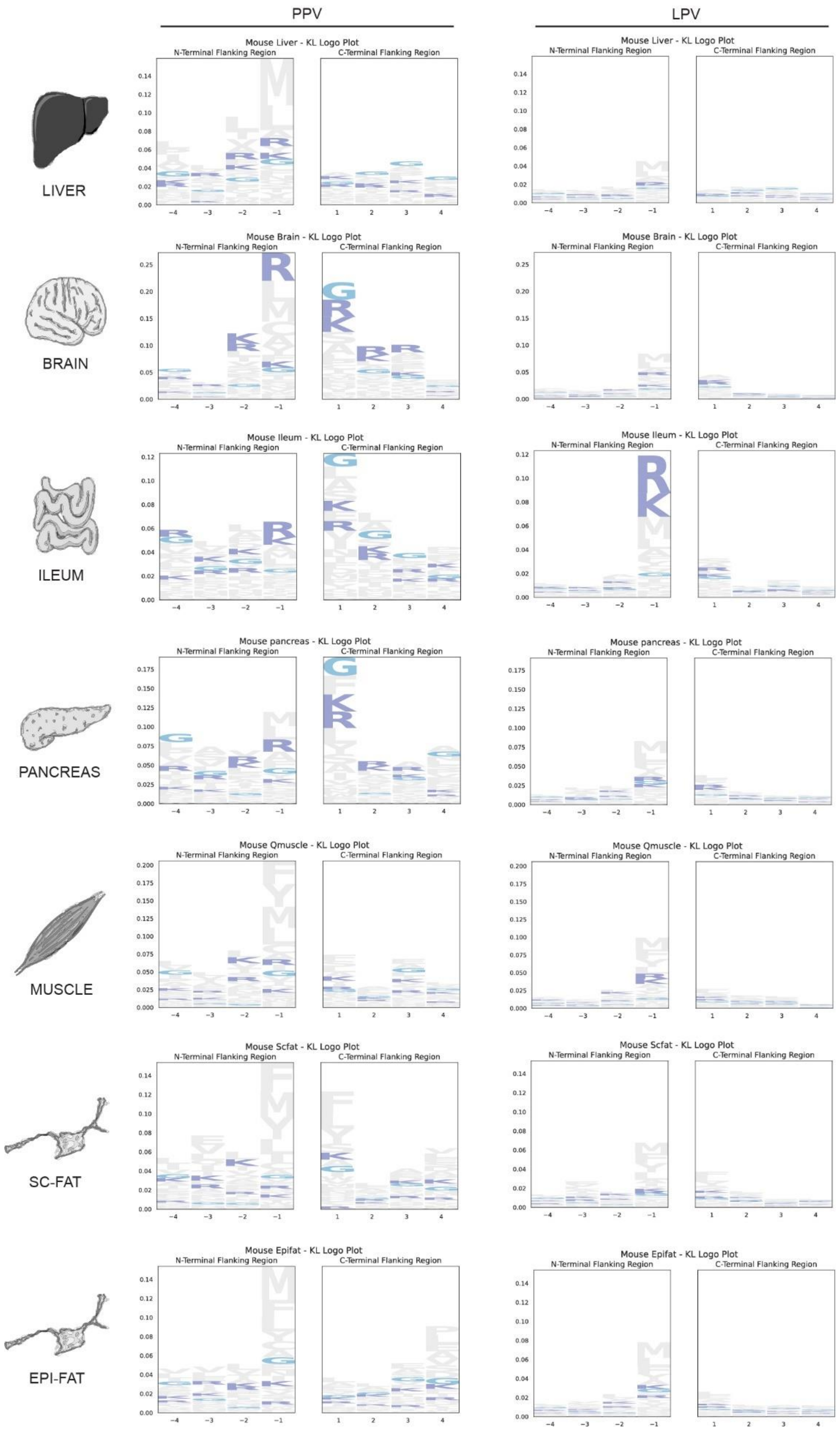

Supplementary Figure 9: See next page for caption.

Supplementary Figure 9: Logo plots of N- and C-terminal flanking regions from two computational models.

Logo-plots of flanking regions surrounding predicted peptides (using Kullback-Leibler divergence) from the PPV model (left) and the LPV model (right). To avoid bias, we removed all known peptides from the analysis in the models and used a pseudo-count of 0.1. Lysine (K) and Argenine (R) are marked in dark blue, and Glycine (G) in light blue. The height of the letters is the frequency of the amino acids in the foreground times the divergence. The Predicted-Peptide-Variant (PPV) model foreground consists of peptides with a score above 0.01, and the background is the 80% lowest scoring PPV peptides. Most tissues show clear enrichment of certain sequence motifs in the N- and C- terminal flanking regions, including di-basic motifs (rich in R and K) and Glycine (G) in position +1 in brain, ileum and pancreas, consistent with known enzymatic processes in these tissues. Muscle and fat tissue peptides on the other hand display markedly different motifs rich in F, M, Y and L. Note that the signals observed are not driven by already known bioactive peptides, since these were specifically removed from the analysis. The Longest-Peptide-Variant (LPV) model (foreground data) is constructed using a set of rules that builds exclusively the longest peptide variant within each cluster[13] and the background is every observed peptide that is not a longest variant (LPV). Data clearly demonstrates that there is little signal (motif enrichment) in simply picking or building the longest possible peptides from observed fragments, consistent with the finding that the PPV clearly outperforms the LPV model in comparisons (see Supplementary Note 1). Tissue images produced using Servier Medical Art (https://smart.servier.com/).

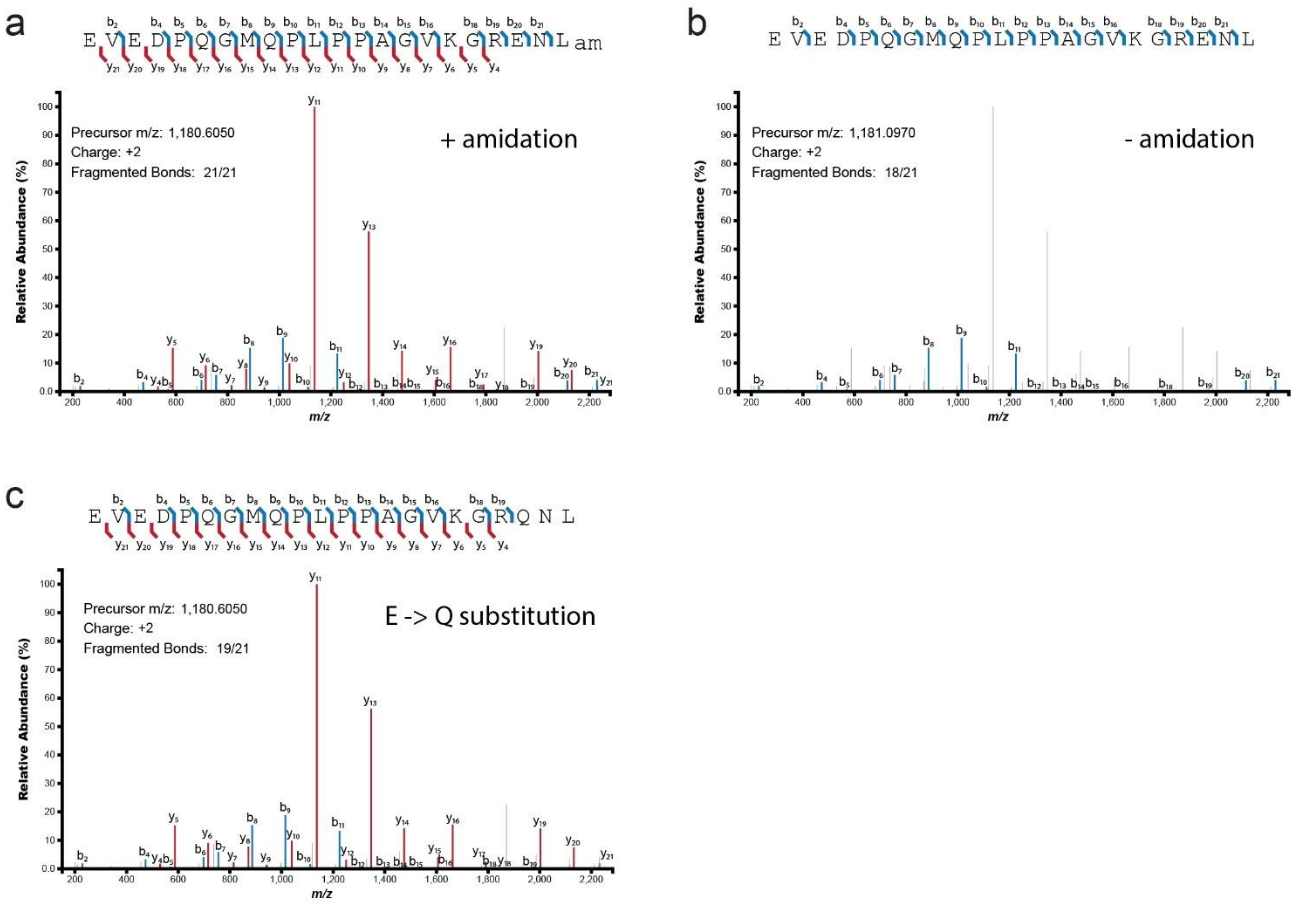

Supplementary Figure 10: MS/MS ion spectra of D-peptide from insulin.

a, y- and b-fragment ions covering the D-peptide sequence. The D-peptide was observed exclusively in pancreas but in all 48 mice with an average precursor mass error of 0.0725 ppm, and an average andromeda score of 226.199. **b**, The reading frame established by the alternative splicing event ends with a Glycine before the stop codon, but the observed peptide ends with a C-terminal amidation group on Leucine confirmed from the y-ion series. The entirety of the y-ion series can only be assigned if the amidation is present in the C-terminal including the y4-ion. **c**, Theoretically the y4-ion could be explained by a Glutamate (RENL) to Glutamine (RQNL) substitution in position 20 (and no C-term. amidation), however the b20 and b21-ions containing the hypothetical E -> Q substitution do not exist. Figures made by the use of the *Interactive Peptide Spectral Annotator*[14].

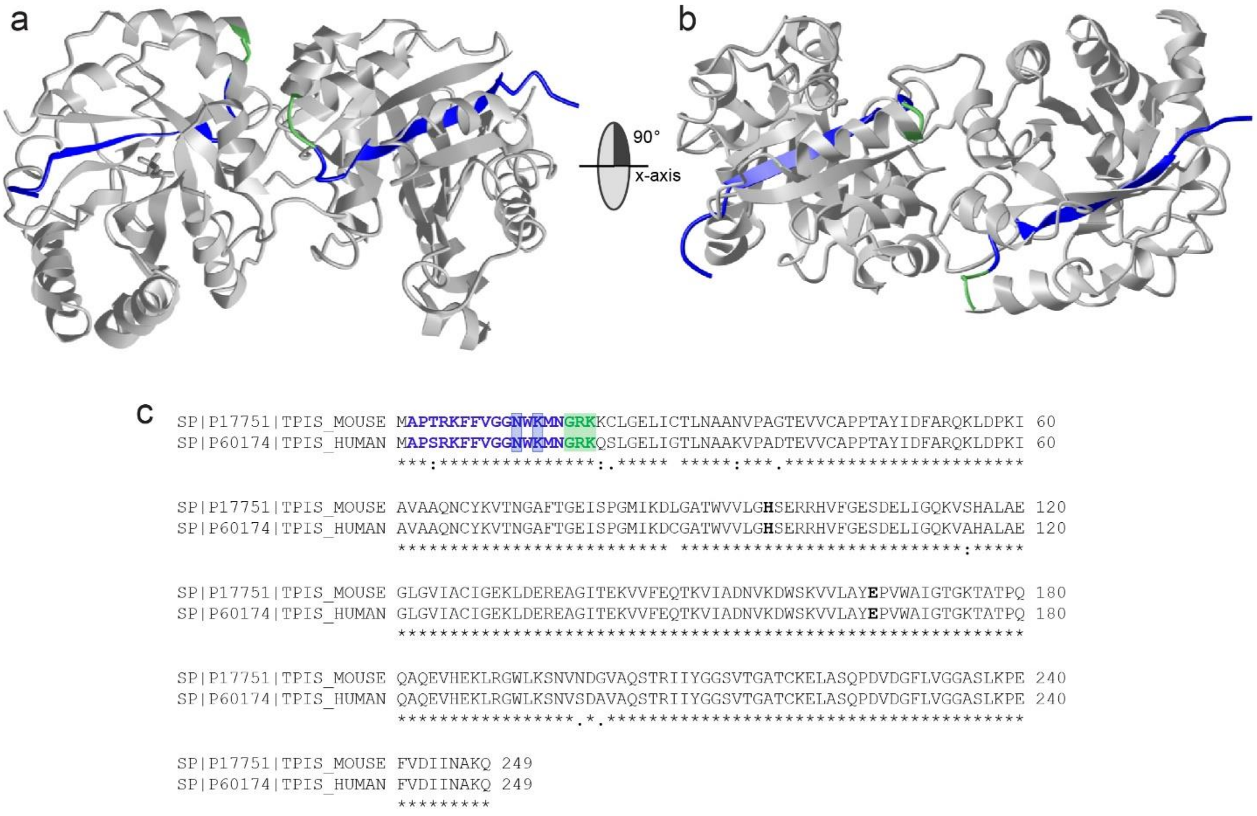

Supplementary Figure 11: Potential bioactive peptide cleaved from Triosephosphate isomerase. **a**, Homodimeric crystal structure (PDB ID: 1HTI) of Triosephosphate isomerase (Tpi1) from human (Uniprot ID: P60174). **b**, Structure rotated 90 degrees on x-axis. Initiator methionine is removed and the β-sheet and the novel APTR-15 peptide (position 2-16) is marked in blue with the GKR containing loop in green. **c**, Amino acid alignment between human and mouse Tpi1 with electrophile (H96) and proton acceptor (E166) in the active site bolded. APTR-15 is mapped to the extended N-terminal isoform in mouse (Uniprot ID: P17751: 52-66) which is indistinguishable to the short isoform in the region of overlap (position 2-16). APTR-15 contains the dihydroacetone phosphate substrate binding site (position N12 and K14) followed by an exposed turn with a GRK canonical cleavage and amidation motif (marked in green). The predicted APTR-15 peptide is amidated, consistent with the Glycine in the +1 position.

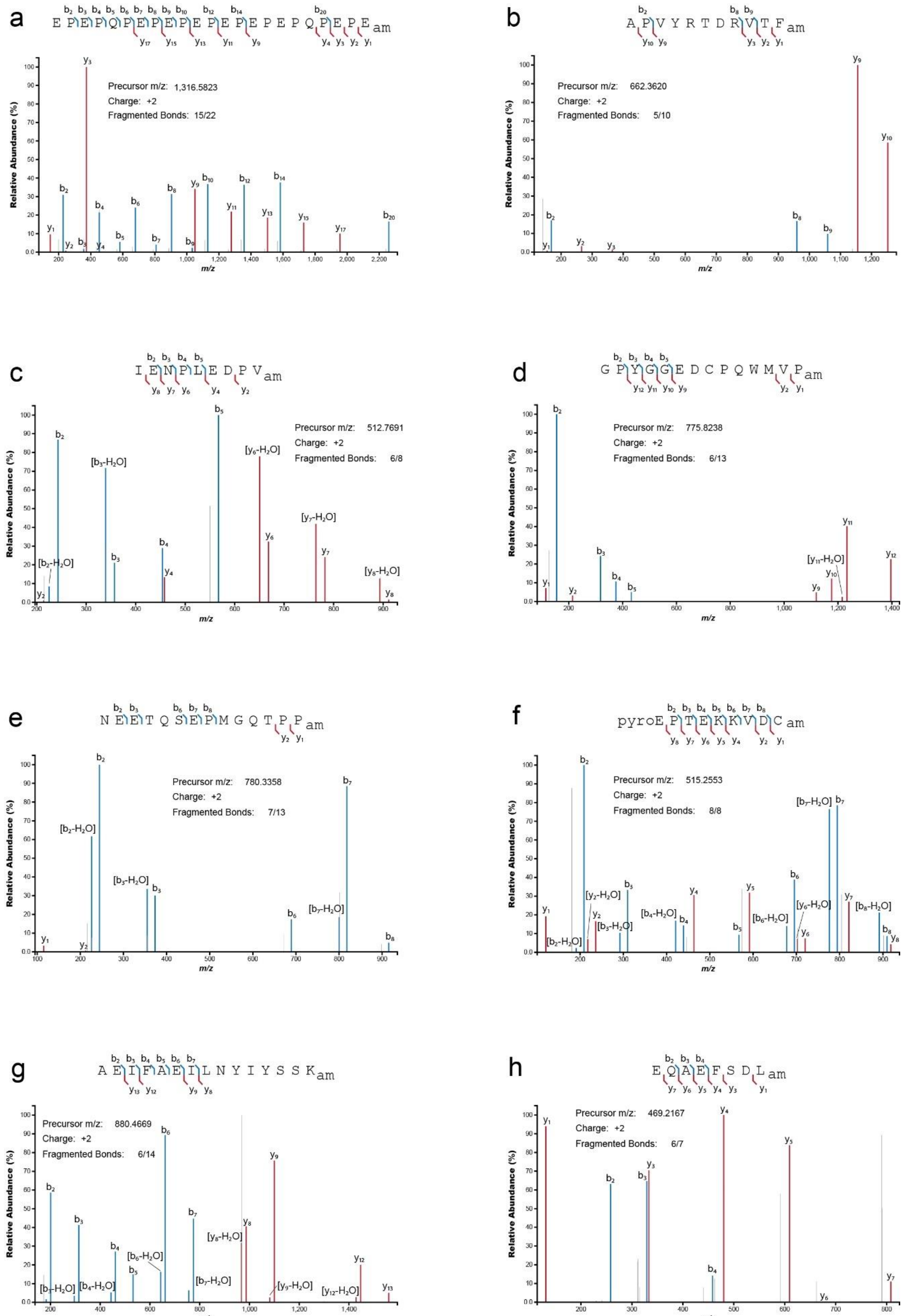

Supplementary Figure 12: See next page for caption.

Supplementary Figure 12: MS/MS ion spectra of non-glycine amidated peptides.
a, y- and b-fragment ions of Gabre (A2AMW3) peptide (327-349) from brain. **b**, y- and b-fragment ions of DysF (Q9ESD7) peptide (1723-1733) from pancreas. **c**, y- and b-fragment ions of Atp6v0a1 (Q9Z1G4) peptide (209-217) from brain. **d**, y- and b-fragment ions of Npepps (Q11011) peptide (531-544) from brain. **e**, y- and b-fragment ions of Inpp5f (Q8CDA1) peptide (997-1010) from liver. **f**, y- and b-fragment ions of Aga (Q64191) peptide (337-345) from pancreas. **g**, y- and b-fragment ions of Zbtb33 (Q8BN78) peptide (75-89) from subcutaneous fat. **h**, y- and b-fragment ions of Syn1 (O88935) peptide (144-151) from brain. Figures made by the use of the *Interactive Peptide Spectral Annotator*[14]. The examples show the presence of an amidation group in y-ions C-terminal positioned from a Glutamic acid (E) or a Aspartic acid (D). The existence of non-glycine amidated peptides are supported by independent observations in rat brain samples[13] and human plasma peptidome samples[15].

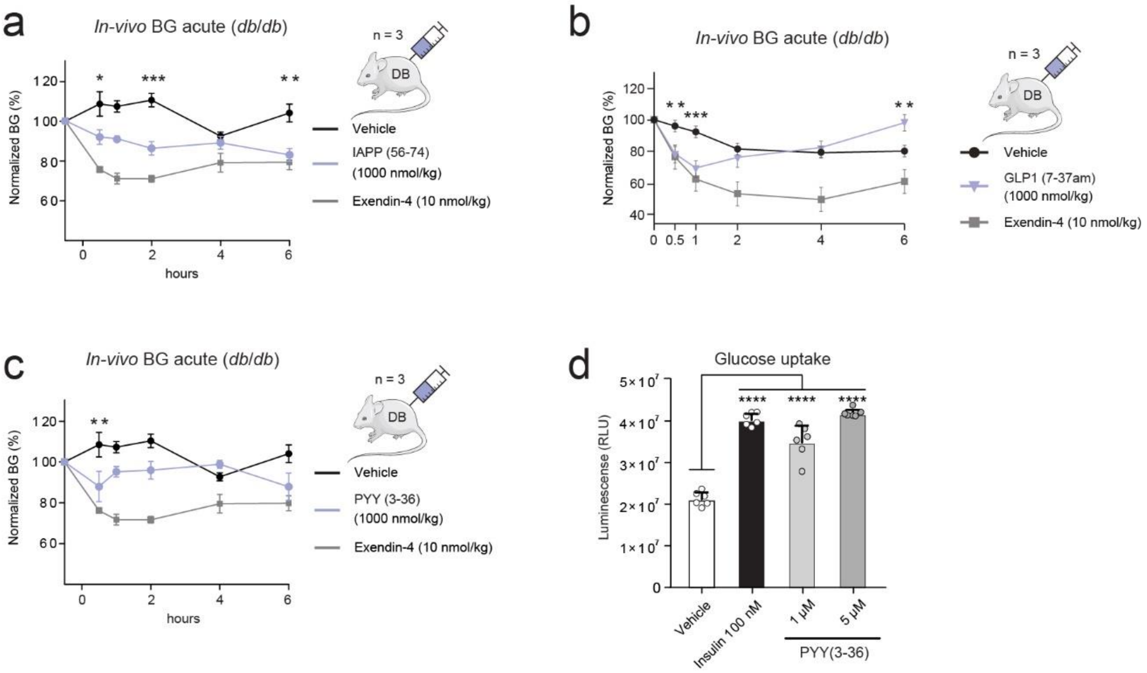

Supplementary Figure 13: Assay controls for acute changes in blood glucose and glucose uptake. **a**, BG mean values ± SEM in n=3 *db/db* mice after Islet amyloid polypeptide (IAPP: 56-74) intraperitoneal injection at 1000 nmol/kg. Baseline blood glucose normalized to 100%. Positive control is the GLP-1 analogue Exendin-4 at 10 nmol/kg. Two-way ANOVA vs. vehicle with post-hoc Dunnett's test, *P* = 0.0483 (0.5h), *P* = 0.0005 (2h), *P* = 0.0036 (6h). **b**, BG mean values ± SEM in n=3 *db/db* mice after GLP1 (7-37am) intraperitoneal injection at 1000 nmol/kg. Positive control is the unnaturally stable GLP-1 analogue Exendin-4 at 10 nmol/kg. Two-way ANOVA vs. vehicle corrected with post-hoc t-test at each time interval. P-values Bonferroni adjusted, *P* = 0.0055 (0.5h), *P* = 0.0004 (1h), *P* = 0.0060 (6h). **c**, BG mean values ± SEM in n=3 *db/db* mice after Peptide Tyrosine Tyrosine (PYY: 3-36) intraperitoneal injection at 1000 nmol/kg. Baseline blood glucose normalized to 100%. Exendin-4 at 10 nmol/kg. Two-way ANOVA vs. vehicle with post hoc Dunnett's test, *P* = 0.0061 (0.5h). **d**, Glucose uptake in 3T3-MBX cells at day 20 (20,000 cells/well) from n=6 independent measurements calculated as mean (luminescence in RLU) ± SD. Cells were treated overnight with diluted PYY (3-36) or with 100 nM insulin as positive control. Unpaired two-sided t-test with *P* < 0.0001 (****). Source data are provided as a Source Data file.

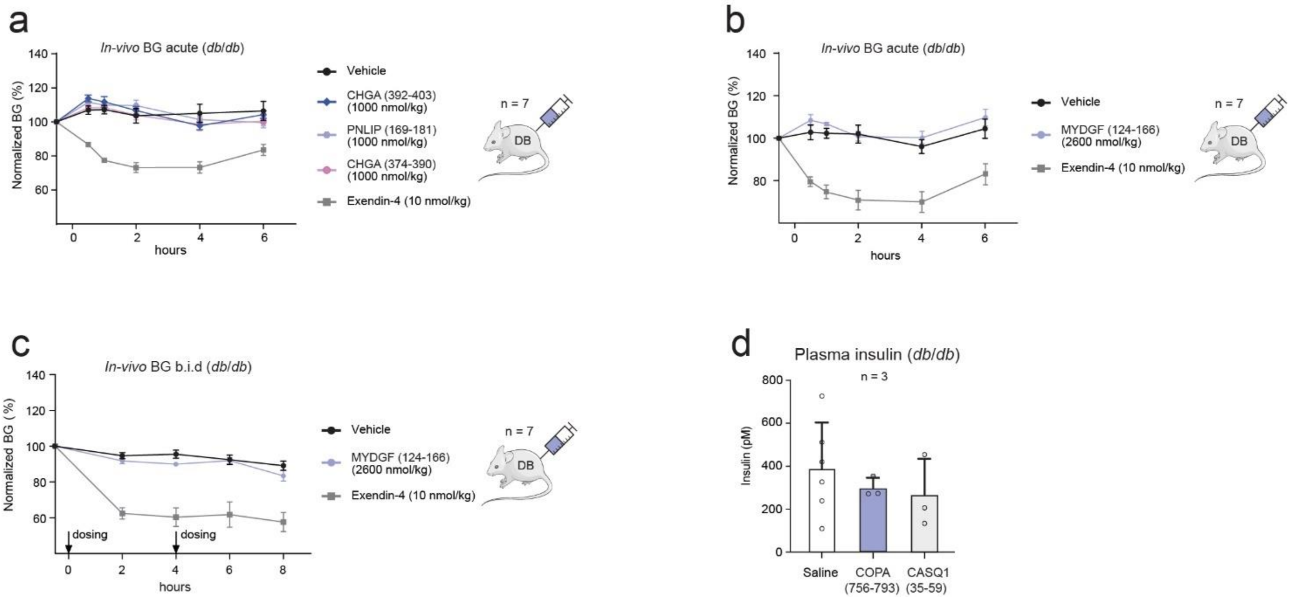

Supplementary Figure 14: Non-functional peptides tested in screening setup.
Non-functional peptides with a PPV score approximately 10x fold lower than the lower threshold (PPV score > 0.01). The MYDGF peptide is produced in *E. coli*, the rest is array synthesized. **a**, BG mean values ± SEM in n=7 *db/db* mice after CHGA (392-403), PNLIP (169-181), CHGA (374-390) intraperitoneal injection at 1000 nmol/kg. Exendin-4 at 10 nmol/kg. Baseline normalized to 100%. Two-way ANOVA with post-hoc t-test of each peptide vs. vehicle with the following P-values: CHGA (392-403); 0.1138 (0.5h), 0.2864 (1h), 0.4826 (2h), 0.0919 (4h), 0.6186 (6h). PNLIP (169-181); 0.2898 (0.5h), 0.5386 (1h), 0.1689 (2h), 0.3987 (4h), 0.1115 (6h). CHGA (374-390); 0.7501 (0.5h), 0.7837 (1h), 0.9457 (2h), 0.1348 (4h), 0.1572 (6h). None of the peptides at any timepoint are significantly different from vehicle. **b**, BG mean values ± SEM in n=7 *db/db* mice after MYDGF (124-166) intraperitoneal injection at 2600 nmol/kg. Exendin-4 at 10 nmol/kg. Baseline normalized to 100%. Two-way ANOVA with post-hoc t-test vs. vehicle with following P-values; 0.2201 (0.5h), 0.3245 (1h), 0.7023 (2h), 0.4222 (4h), 0.2752 (6h). To all timepoints not significant. **c**, BG mean values ± SEM in n=7 *db/db* mice by twice daily MYDGF (124-166) peptide dosing at 2600 nmol/kg. Two-way ANOVA with post-hoc t-test vs. vehicle with following P-values; 0.3470 (0.5h), 0.3470 (1h), 0.3470 (2h), 0.0755 (4h), 0.8757 (6h). To all timepoints not significant. **d**, Plasma insulin (pmol/L) mean values ± SD in n=3 *db/db* mice 6 hours after COPA (756-793) and CASQ1 (35-59) dosing at 354 nmol/kg and 402 nmol/kg. Saline control is n=6. Unpaired two-sided t-test, with P = 0.5166 for COPA, and P = 0.4097 for CASQ1 for difference in mean to saline (not significant). Randomly selected non-functional peptides does not produce any read-out in the *db/db* animal model. Source data are provided as a Source Data file.

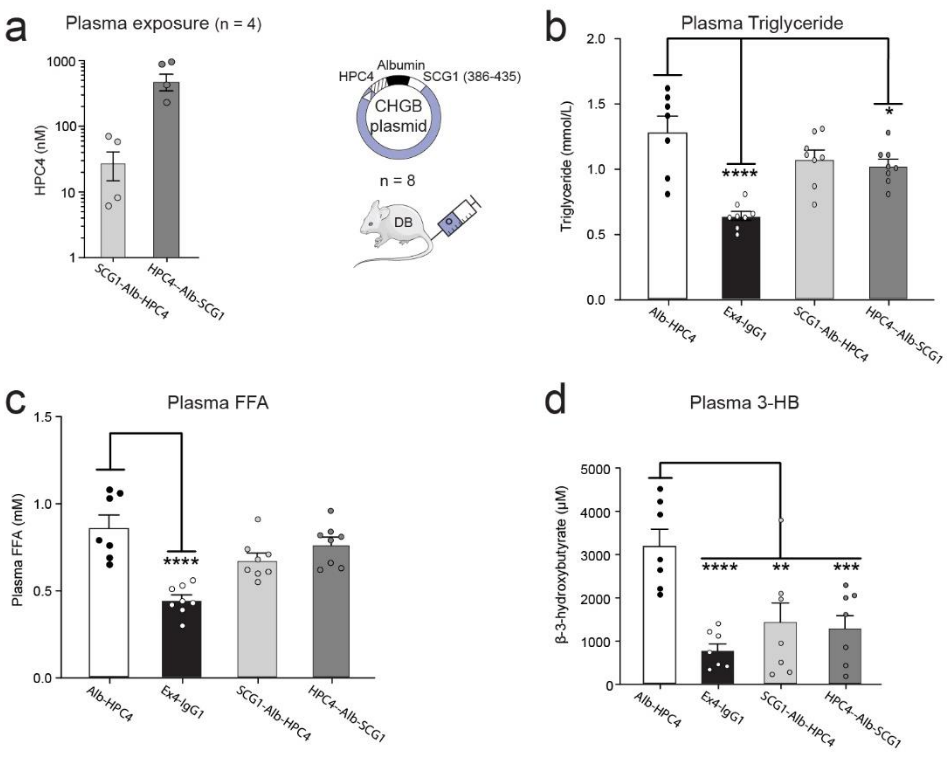

Supplementary Figure 15: Hydrodynamic gene delivery of SCG1 peptide NHPD-50 in *db/db* mice.
a, Plasma exposure of N- and C-terminal albumin protracted SCG1 (386-435) after hydrodynamic gene delivery. Mean values ± SEM in n=4 *db/db* mice of HPC4 in plasma at day 7. **b**, Mean values ± SEM (mmol/L) of plasma triglycerides from albumin protracted SCG1 (386-435) constructs in n=8 animals in each group. Albumin-HPC4 is negative control, and Exendin-4-IgG1 positive control. One-way ANOVA vs. control with post hoc Dunnett's multiple comparisons test, $P < 0.0001$ (Ex4-IgG1), $P = 0.0486$ (HPC4-Alb-SCG1). **c**, Mean values ± SEM (mmol/L) of plasma free-fatty acids from albumin protracted SCG1 (386-435) constructs in n=8 animals. One-way ANOVA with post hoc Dunnett's test, $P < 0.0001$ (Ex4-IgG1). **d**, Mean values ± SEM (µmol/L) of plasma 3-hydroxybutyric acid in n=8 animals. One-way ANOVA with post hoc Dunnett's test, $P < 0.0001$ (Ex4-IgG1), $P = 0.0011$ (SCG1-Alb-HPC4), $P = 0.0004$ (HPC4-Alb-SCG1). Source data are provided as a Source Data file.

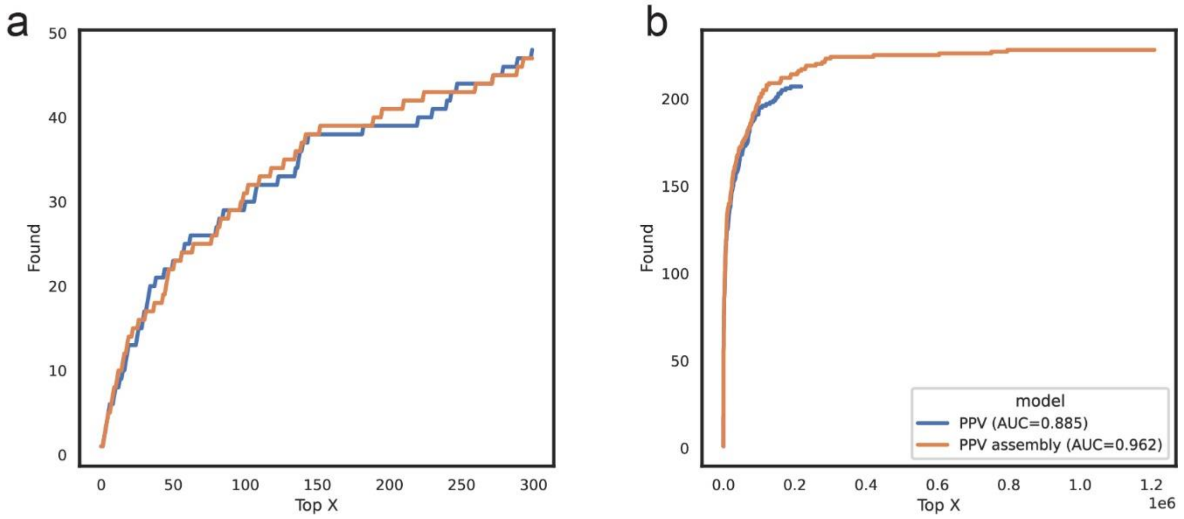

Supplementary Figure 16: Comparing PPV model with the PPV-assembly model.
a, Number of known training peptides identified as a function of rank (based on prediction score) for each method. PPV model (blue), PPV-assembly (orange) on top300 highest scoring predictions. **b**, The assembly model artificially increase the data to 1.2 million peptides and ultimately assemble more known annotated peptides. For details on the individual models, see the Supplementary Note 1.

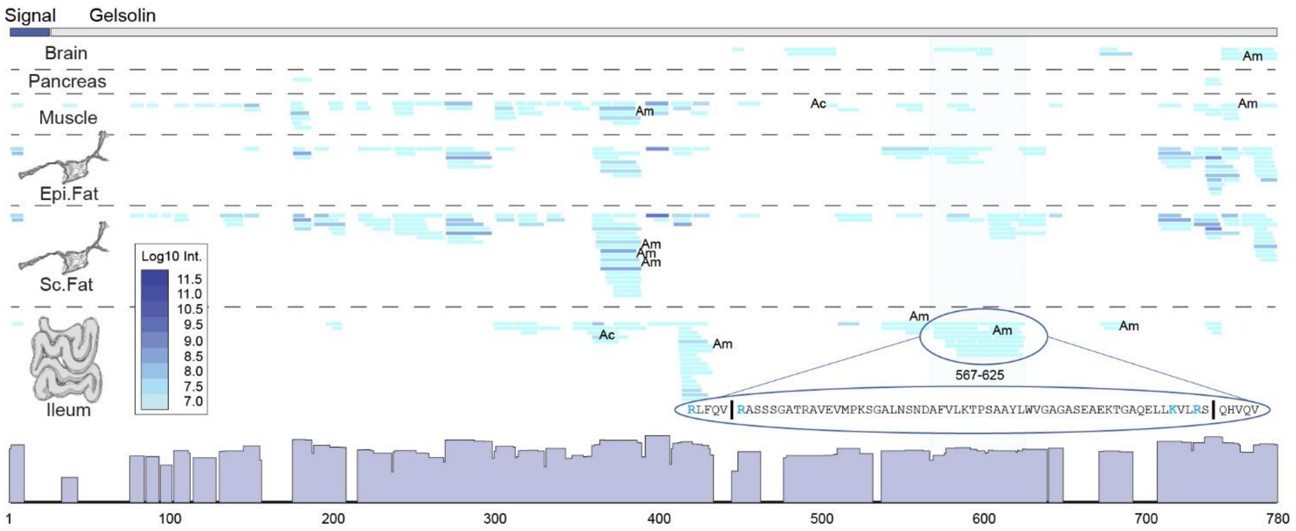

Supplementary Figure 17: Gelsolin peptides and PPV-assembly.

Observed Gelsolin peptides mapped onto the Gelsolin protein backbone (Uniprot ID: P13020). Peptides are colour coded according to abundance (log10 intensity). The PPV-assembly model assembles breakdown fragments in the highlighted region into a predicted peptide (spanning 567-625) which is subsequently found to induce glucose uptake and insulin secretion *in-vitro* (see main paper).

Supplementary Note 1

Last revised: 9 October 2022

Contents

1 Introduction **3**

2 Introducing the data **4**
2.1 MS data . . . 5
2.2 Positive training set . . . 5
2.3 Negative training set . . . 6

3 Feature Engineering **7**
3.1 Mathematical Definitions used in multiple MS features . . . 8
3.2 Intensity: start and stop . . . 10
3.3 Bool: First and Last . . . 11
3.4 Frequency: Start and Stop . . . 12
3.5 Frequency: Observed . . . 13
3.6 Frequency: Sample . . . 14
3.7 Frequency: PTMs . . . 15
3.8 Frequency Bond . . . 16
3.9 MS ladder . . . 17
3.10 MS Intensity: penalty start/stop . . . 18
3.11 MS Bool: observed . . . 18

4 Machine Learning **19**
4.1 Data Partitioning . . . 20
4.1.1 Nested Cross Validation . . . 20
4.1.2 Stratified 5 Group Split . . . 20
4.2 Model Specification . . . 20
4.3 Model Evaluation . . . 22
4.3.1 Comparing to PeptideRanker . . . 23
4.3.2 Performance evaluation based on mislabeled peptides . . . 24
4.3.3 Enrichment of known cleavage sites and peptides from secreted proteins . . . 24
4.4 Ablation Experiments . . . 26
4.4.1 Oversampling of Positives . . . 26
4.4.2 Random undersampeling . . . 26
4.4.3 Removal of Long Peptides . . . 26

5 The Assembly-PPV model **29**
5.1 Motivation . . . 30
5.2 Assembly of longer peptides . . . 30
5.2.1 Model design and training . . . 31
5.2.2 Model parameters . . . 31
5.2.3 Model performance . . . 31
5.3 Limitations and Future research . . . 32

Chapter 1

Introduction

This document serves as technical note 1 of the manuscript: *Combining mass spectrometry and machine learning to discover bioactive peptides, Christian T. Madsen, Jan C. Refsgaard, Felix Teufel, Sonny K. Kjærulff, Zhe Wang, Guangjun Meng, Carsten Jessen, Petteri Heljo, Qunfeng Jiang, Xin Zhao, Bo Wu, Xueping Zhou, Yang Tang, Jacob F. Jeppesen, Christian D. Kelstrup, Stephen T. Buckley, Søren Tullin, Jan Nygaard-Jensen, Xiaoli Chen, Fang Zhang, Jesper V. Olsen, Dan Han, Mads Grønborg, Ulrik de Lichtenberg.*

It provides the details on the data sets used (Chapter 2), the feature engineering (Chapter 3), the model specification and training, model performance evaluation (Chapter 4) and the extended PPV-assembly model (Chapter 5).

Chapter 2

Introducing the data

We first introduce the data available for training a method to recognize real peptides.

2.1 MS data

Mass Spectrometry (MS) analysis was performed on a total of 336 unique samples: 4 groups of mice (HFD, LFD, DB and WT) with 12 individual mice in each group (replicates). From these, we acquired MS data from seven distinct tissues: liver, muscle, intestine (ileum), brain, pancreas, epididymal fat and subcutaneous fat. The MS analysis identifies the unique peptide sequences present in the sample (by comparing the mass-charge values to a database of theoretical fragments) and provides an estimate of the abundance of the peptide in that particular sample. The abundance is also sometimes referred to as the intensity. Although very short bioactive peptides are known to exists, we did (for technical reasons) only included peptides of length 7 or longer when searching the MS spectra. For the sake of training the model, for all but one feature, we compute a robust estimate of the abundance of each observed peptide in each tissue by summing the abundance values from all 48 samples (representing different mice and experimental conditions). The MS-derived features used for training and prediction (see below) are therefore computed from the data for each peptide in each tissue.

2.2 Positive training set

The model training step requires a set of labeled data with positive and negative examples. To build the set of positive training examples, we extracted known mouse peptides annotated in Uniprot (`Version 26 Jan 2017`), SwePep and NeuroPep with amino acid sequence and relative position in the parent protein (counting positions from the N-terminus to the C-terminus). Examples include both known bioactive peptides, like Glucagon and GLP-1, and annotated propeptides and structural peptides, such as the C-terminal peptide of NPY. We noticed many cases in which the structural peptides and propeptides were clearly visible in MS, and therefore included them as positive examples to train a model which would recognize the patterns characteristic of peptides produced by proteases and convertases, regardless of whether these peptides are bioactive or not.

The training set contained a total of 275 annotated mouse peptides and 795 propeptides (See Supplementary Tabel 3). Of these, 76 peptides and 20 propeptides were observed in our MS data as full length perfect matches in at least one tissue. Since many of these 96 peptides and propeptides were observed in multiple tissues, the final positive training set consists of 207 cases of MS-observed known annotated peptides or propetides, with 173 cases corresponding to known peptides and 34 cases corresponding to known propeptides.

The longest MS-observed sequences matching a known peptide/propeptide were 42 amino acids (example: Intestinal peptide PHI-42) but across the whole MS data set we did observe sequences up to 79 amino acids in length (although these did not match known annotated peptides or propeptides). Peptide spectral matching works by assigning peptide fragments to peptide sequences, the number of possible fragments correlates with the peptide length, thus peptides shorter than 7 are usually indistinguishable from noise and therefore excluded from the search space a prori. This means that by design, short peptides like Met-enkephalin (length 5) were excluded from our analysis. Curiously, however, the protein precursor which produces the very short Met-enkephalin (known as Proenkephalin-A or PENK) also produces a much longer peptide known as PENK(143-184) which

happens to be one of the longest known peptides observed.

The **final positive training set** therefore consists of **207** cases of peptides/propeptides of length 7 to 42 amino acids. Some were observed with N-terminal acetylation or C-terminal amidation but this information is only indirectly used when deriving the features (see later).
The positive training data is available as Supplementary Data 3 to the main paper.

2.3 Negative training set

Since peptide databases do not provide negative annotations (e.g. that a particular fragment of a particular protein is not a bioactive peptide), we instead relied on the assumption that the vast majority (best guess, more than 99%) of the 150.150 unique sequences observed in our MS data set correspond to degradation fragments of normal proteins or peptides which are not processed purposely by the cell into bioactive peptides. For instance, we observe thousands of fragments of Albumin in each of the seven tissues which is hardly surprising, since this is the most abundant protein in blood. Some of the 150.150 sequences were observed in multiple tissues, resulting in 217,695 total MS observations. 3179 of these were longer than the longest positive training example of 42 amino acids (length of 43-79, See Section 4.4.3). We therefore labeled all observed peptide sequences of length 7-42 and not belonging to the positive set as negative. The negative set thereby also includes all the shorter or longer fragments of the known annotated peptides.

The **final negative set** therefore consists of **214,516** MS observations of peptide sequences (7-42 amino acids in length), none of which are exact matches to annotated peptides or propeptides.

Chapter 3

Feature Engineering

Given the small set of positive training examples available we did not attempt approaches that would learn features from the data (e.g. as in deep learning on images) but instead used classical feature engineering to construct features we believe would capture informative aspects of the data. Training on the data later revealed which were the more information rich. We explored only Mass spectrometry derived features (using the MS data patterns)
For the sake of this analysis, we can think of the peptides as unique amino acid sequences observed with a particular abundance (e.g. 10^8) in each tissue (or not at all in some tissues). For example, the known bioactive peptide GLP1 has the sequence HAEGTFTSDVSSYLEGQAAKEFIAWLVKGR and is observed in intestinal tissue (Ileum) where it is also known to be produced. It corresponds to position 98-127 in its parent protein (pre-proglucagon, P55095). The parent protein is 180 amino acids long and can thus be represented as a vector of length 180 onto which all the fragments observed can be mapped, including our example GLP1 (position 98-127). The MS-features are all derived as a single numerical value assigned to each individual peptide (in each tissue) and calculated by aggregating and comparing properties across positions in the parent protein (index i) and across peptides mapping to the parent protein. Most features are extracted in two steps:

- A property is calculated per position (using the index i) in the parent protein resulting in a vector of numbers.
- For each peptide, the above feature vector is used to extract a scalar number, which is assigned to the peptide. This is written as $x_{\text{feature name},j}$. In the code (see below) this is a pandas DataFrame, where the feature column has name close to "feature name" subscript of x.

3.1 Mathematical Definitions used in multiple MS features

The data set has many dimensions, as there are multiple tissues, multiple proteins per tissue, multiple clusters of peptides per protein, multiple peptides per cluster with observations in multiple samples representing different conditions and replicates. In the following, a cluster is defined as a region of the protein, e.g. position 98-127, where one or more peptides cover the region. See the Figures in the main article to get a feel for what the clusters typically look like.

For each tissue, protein, cluster combination we extracted features as described below. Here, 'tissue', 'protein' and 'cluster' subscripts have been omitted to keep the notation less confusing. As mentioned, most features are extracted by iterating over the different peptides (index j) which span a particular position (index i) along the protein backbone to form a positional feature vector which is then converted into a single numerical value for each individual peptide (index j). For all but 1 feature we thus initially sum across all 48 samples to create $X_{i,j}$ which contains the (total summed) mass spectrometer recorded intensity (or abundance) of peptide j at all positions i covered by the peptide and 0 at all other positions j, i. Based on that, we defined indexes and boundaries as follows:

$$\begin{aligned}
&\text{let } i \text{ be the amino acid position in the peptide cluster}\\
&\text{let } N \text{ be the number of amino acids in the peptide cluster}\\
&\text{let } j \text{ be the peptide index in the peptide cluster}\\
&\text{let } M \text{ be the number of peptides observed in the peptide cluster}\\
&\text{let } k \text{ be the sample index}\\
&\text{let } O \text{ be the number of samples}
\end{aligned} \tag{3.1}$$

Allowing two functions to be defined that map the peptides from peptide indexes j to coordinates i:

$$\begin{aligned}&\text{let } p_{start}(j) \text{ be a function that returns the first index } (i) \text{ of peptide } j\\&\text{let } p_{stop}(j) \text{ be a function that returns the last index } (i) \text{ of peptide } j\end{aligned} \tag{3.2}$$

The peptide abundance (or intensity) was then defined as:

$$\begin{aligned}&\text{let } I = \text{be a matrix of shape } [M, O]\\&\text{let } I_{j,k} \text{ be the normalized intensity recorded by the mass spectrometer}\\&\qquad \text{for a peptide j in sample k}\end{aligned} \tag{3.3}$$

From the abundance/intensity I we created the tensor Z where:

$$\begin{aligned}&Z \text{ is a tensor of shape } [N, M, O]\\&Z_{i,j,k} = \begin{cases} I_{j,k}, & \text{if } p_{start}(j) \leq i \leq p_{stop}(j),\\ 0, & otherwise\end{cases}\end{aligned} \tag{3.4}$$

For most features, we instead start from the combined (summed) abundance of each peptide which removes the sample index k to create an intensity matrix $X_{i,j}$ which defines the intensity of each peptide (j) at each position (i):

$$\begin{aligned}&\text{let } X \text{ be a matrix of shape } [N, M]\\&\text{let } X_{i,j} = \sum_{k=1}^{k=O} Z_{i,j,k}\end{aligned} \tag{3.5}$$

This matrix X is depicted for most of the MS-derived features (Figures between 18-22). Because many of the engineered features are based on comparing the abundance of a given peptides in a cluster to that of all other peptides in that region of the protein backbone, we created histogram (H_i) vectors where we sum over the individual peptides j overlapping position i:

$$\begin{aligned}&\text{let } H \text{ be a vector of length } N\\&\text{let } H_i = \sum_{j=1}^{j=M} X_{i,j}\\&\text{let } H_{am} \text{ be a vector of length } N\\&\text{let } H_{am,i} = \begin{cases} \sum_{j \subset amidated} X_{i,j}, & \text{if } p_{start}(j) = i\\ 0, & otherwise\end{cases}\\&\text{let } H_{ac} \text{ be a vector of length } N\\&\text{let } H_{ac,i} = \begin{cases} \sum_{j \subset acetylated} X_{i,j}, & \text{if } p_{stop}(j) = i\\ 0, & otherwise\end{cases}\end{aligned} \tag{3.6}$$

Finally, since we engineered a few features which express the number of samples in which the peptide was observed, we also defined P_j as follows:

$$\begin{aligned}&\text{let } P \text{ be a vector of length } M\\&\text{let } P_j = \sum_{k=0}^{O} I_{j,k}\end{aligned} \tag{3.7}$$

Each of the individual features that we engineered or predicted are explained below.

3.2 Intensity: start and stop

This feature `Intensity Start` expresses the change in abundance (or intensity) observed across all peptides when comparing the first amino acid position (the N-terminal of the peptide) to the previous position, as illustrated in Supplementary Figure 18a.

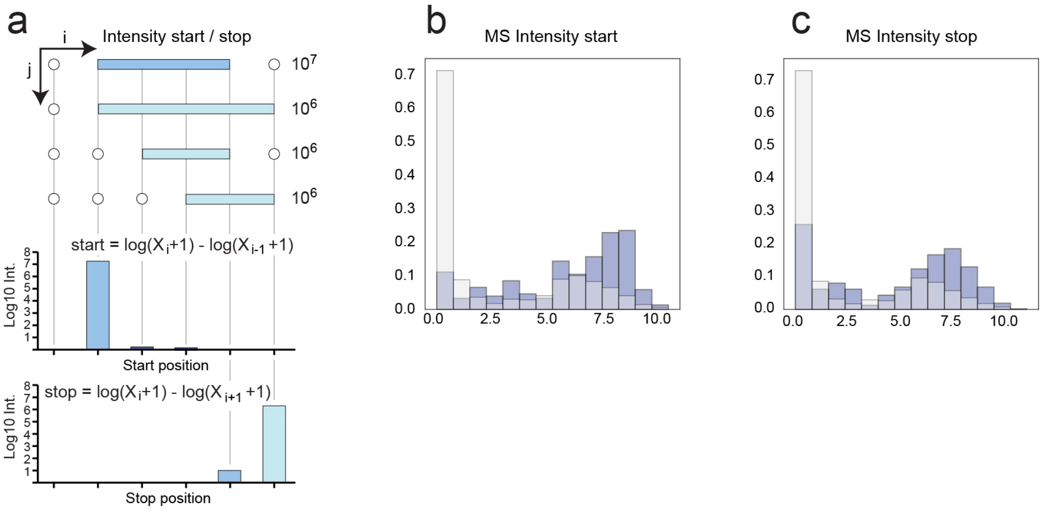

Supplementary Figure 18: a, illustration of how the features is derived. b and c, distribution of feature values for positive (blue) and negative (grey) training examples for Intensity start and Intensity stop, respectively.

At each position along the protein precursor, we first sum the abundance of all observed peptides (Supplementary Figure 18a top) which overlap with this position to produce an abundance histogram or vector (Supplementary Figure 18 bottom). For a given observed peptide, we then define `Intensity Start` as the log_{10} of the difference between the summarized abundance value at the position corresponding to the first amino acid in the peptide and that of the preceding position. This feature becomes large when the first position corresponds to the first position of a high abundance peptide or a peptide cluster, as illustrated in Supplementary Figure 18a. `Intensity Stop` is the equivalent feature to capture if a peptide aligns with the C-terminal end of a cluster.

The features are calculated as:

$$
\begin{aligned}
\text{let } \Delta start_i &= \begin{cases} log_{10}(H_i - H_{i-1}) + 1) & \text{if } H_{i-1} \leq H_i \\ 0, & \text{otherwise} \end{cases} \\
\text{let } \Delta stop_i &= \begin{cases} log_{10}(H_i - H_{i+1} + 1), & \text{if } H_{i+1} \leq H_i \\ 0, & \text{otherwise} \end{cases} \\
x_{\text{Intensity start},j} &= \Delta start_{p_{start(j)}} \\
x_{\text{Intensity start},j} &= \Delta stop_{p_{stop(j)}}
\end{aligned} \tag{3.8}
$$

The abundance of peptides in MS follows an instrument dependent distribution which can be described as a skewed and truncated log normal distribution. Below a certain threshold, we can no longer reliably detect the peptides and this creates large differences in abundance for those peptides close to the detection limit. In our samples, the most abundant peptides are around 10^{10} while the least abundant are around 10^6. Those which are even less abundant, however, end up being set to 0 in the analysis (not detected). This creates an undesired jump in the values of the features when computing the abundance difference between a peptides end-position and the previous position. If we imagine a highly abundant peptide with 10^{10} starting at position 50 in the parent protein and compare that to a low abundance fragment (10^6) which overlaps with the position before (position 49), then the abundance difference (and Intensity Start value) would be $\log 10^{10} - \log 10^6 = 4$. If, on the other hand, the low frequency peptide was below the detection limit its value would be zero resulting in a difference of 10, which is a much larger difference, even though the biological scenario is very similar. To put it in another way: it only takes a very small fraction of uncleaved peptides at an otherwise very clear cleavage position to dramatically reduce the feature value of Intensity Start, compared to the cases where the uncleaved peptide is below detection limit.

One possible solution to this problem would be to impute a number close to the instrument detection limit (setting all values below detection to this value). We instead chose to let the model learn from the data by including two additional parameters (Bool start and Bool stop). The regression coefficient of those two input parameters thus act as imputation in the model.

As can be seen from Supplementary Figure 18b and c, the Intensity start and Intensity stop features are distributed such that high values are mostly seen for the positive examples (the real peptides), indicating that this parameter does indeed contain information useful for predicting class membership.

3.3 Bool: First and Last

The feature `Bool First` is designed to capture if a particular peptide's N-terminus aligns with the start of a peptide cluster. The logic behind it is that the known bioactive peptides often manifest as distinct clusters where the real peptide is observed as the longest fragment (but not always the most abundant one). Knowing if a peptide is "first" (or "last") can also act as imputation parameters to balance out the detection limit problem that the Intensity Start and Intensity Stop features suffer from. `Bool First` is set to 1 (True) when no other overlapping peptides exist (in this parent protein) that start upstream of the peptide in question. It is illustrated in Supplementary Figure 19a where the first two peptides (from the top down) would be assigned the value 1 for the feature `Bool First`, because there are no peptides upstream of them that overlap with them.
Vice versa, the feature `Bool Last` is set to 1 (True) when no other overlapping peptides exist (in this parent protein) that end beyond the C-terminus of the peptide in question. In other words, this parameter is True if the peptide aligns with the C-terminal end of a peptide cluster, as illustrated in Supplementary Figure 19a where the second and forth peptide would have the value 1 (True) for this feature.
The two Bool features can, as mentioned above, be thought of as the imputation values for the `Intensity Start` and `Intensity Stop` features explained above. Formally what we do is:

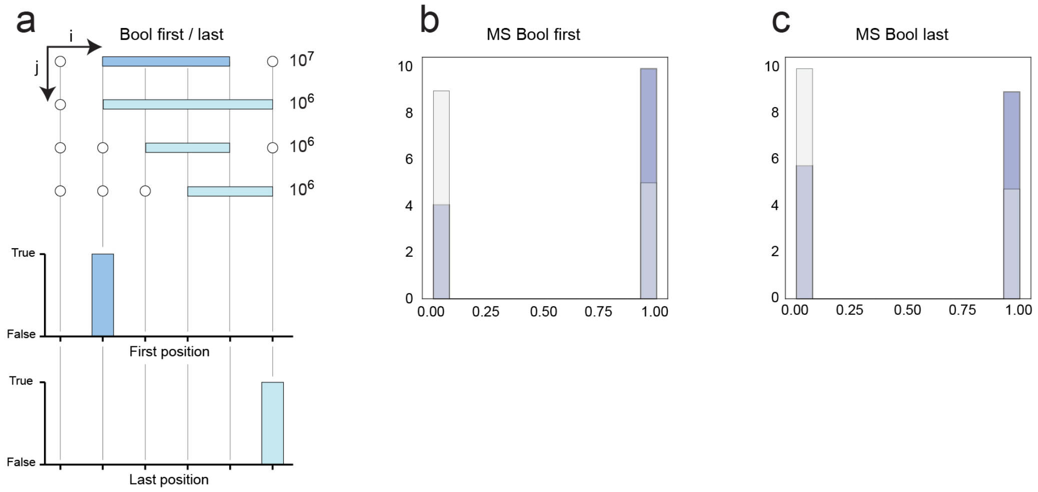

Supplementary Figure 19: a, illustration of how the features is derived. b and c, distribution of feature values for positive (blue) and negative (grey) training examples for Bool first and Bool last, respectively.

$$\text{let } Bool\_First_i = \begin{cases} 1, & \text{if } i = 1 \text{ or } \sum_{j=1}^{j=M} X_{i-1,j} \\ 0, & \text{otherwise} \end{cases}$$
$$\text{let } Bool\_Last_i = \begin{cases} 1, & \text{if } i = N \text{ or } \sum_{j=1}^{j=M} X_{i+1,h} \\ 0, & \text{otherwise} \end{cases} \tag{3.9}$$

Supplementary Figure 19b and c show the distributions of positive and negative examples. As expected, the feature does seem to indicate that the real peptides (positive examples) are more often aligned with the start and end of a cluster.

3.4 Frequency: Start and Stop

These two parameters capture the frequency (or fraction) of peptides which start (or stop) at a given position relative to those that overlap the position. The calculation is done at the level of peptide abundance, and not as a simple counts, to take into account the large differences in abundance of the peptides. The Frequency Start feature is high for peptides whose start position is not "contradicted" by the observation of other overlapping peptides. It can be thought of as expressing if a particular peptide fits with the start of a cluster or is observed in isolation. Frequency Stop is the equivalent but at the C-terminal end of the peptide. Both features would be high in cases where a high abundance peptide spans an entire cluster (of shorter sub-fragments). Similarly it is high when a single distinct peptide is observed without sub-fragments.
Supplementary Figure 20a depict how the two features are extracted.
The formal mathematical expression is:

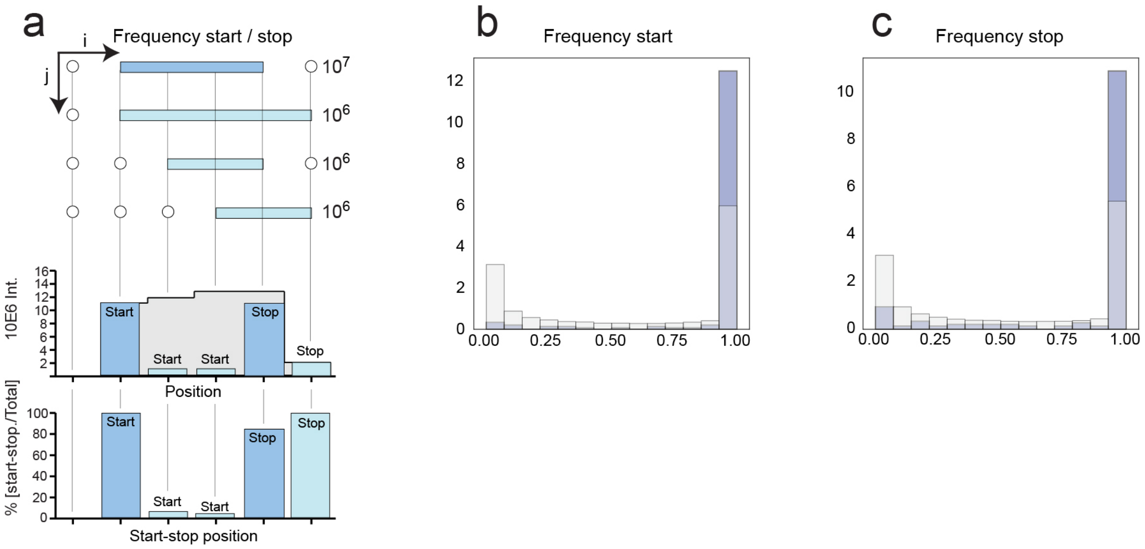

Supplementary Figure 20: a, illustration of how the features is derived. b and c, distribution of feature values for positive (blue) and negative (grey) training examples for Frequency start and Frequency last, respectively.

$$
\begin{aligned}
\text{let } start_i &= \sum_{j=1}^{j=M} \begin{cases} X_{i,j}, & \text{if } p_{start}(j) = i \\ 0, & otherwise \end{cases} \\
\text{let } stop_i &= \sum_{j=1}^{j=M} \begin{cases} X_{i,j}, & \text{if } p_{stop}(j) = i \\ 0, & otherwise \end{cases} \\
x_{\text{Frequency start},j} &= \frac{start_{p_{start(j)}}}{H_i} \\
x_{\text{Frequency stop},j} &= \frac{stop_{p_{stop(j)}}}{H_i}
\end{aligned} \tag{3.10}
$$

3.5 Frequency: Observed

This feature expresses the abundance of a particular peptide relative to the total abundance of all peptides which span the region of the peptide in question. It can be thought of as the percentage of the signal coming from a particular peptide across the region spanned by that peptide. The maximum possible value of 1 occurs if a peptide has no overlapping peptides or shorter sub-fragments observed. Supplementary Figure 21a depict how the features are extracted.
The summarized abundance of the peptide j (across all samples in a tissue) is multiplied by the length of the peptide and divided by the sum of the total abundances (across all peptides) along the length of the peptide:

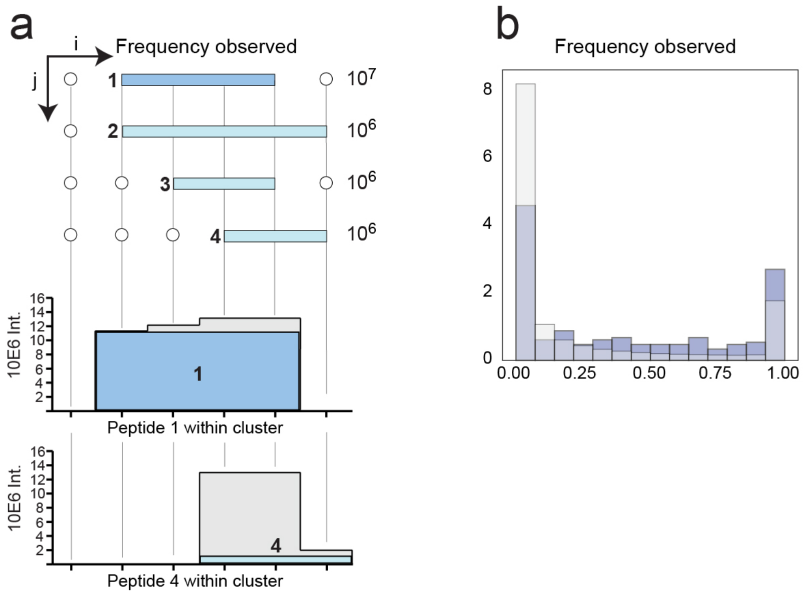

Supplementary Figure 21: a) the abundance of the peptide times it's length is compared the sum of all peptides who intersect with it, middle: as peptide 1 is very intense it's abundance becomes close is 1, lower: where peptide 4 is responsible for very little of the abundance b) histogram of feature distribution, colored blue for the positive set and grey for the negative.

$$x_{\text{Frequency observed},j} = \frac{A_j \times length_j}{\sum_{i=p_{start}(j)}^{p_{stop}(j)} H_i}$$
$$\text{Where } A_j = \sum_{i=p_{start}(j)}^{p_{stop}(j)} I_{j,k} \tag{3.11}$$
$$\text{Where } length = p_{stop}(j) - p_{start}(j) + 1$$

where A_j is the abundance (or intensity) of peptide j summarized across all samples in a tissue and $length_j$ is the length of the peptide j. H_i is the sum of the abundance values for all peptides which overlap position i. The denominator sums this quantity over the length of the peptide j. As can be seen in Supplementary Figure 21b, the value of the feature seem to be positively correlated with being a real peptide. The distribution thus indicates that this feature could contribute positively to identifying the most abundant peptide in a cluster and that this would often be the real bioactive peptide.

3.6 Frequency: Sample

This features expresses how consistently a particular peptide is observed across samples (conditions and replicates), regardless of its absolute abundance in these samples. It should thus be expected to be highly correlated with the previous features but will additionally capture information on peptides which are perhaps not the most abundant but nonetheless consistently found in a particular tissue.

Supplementary Figure 22a depict how the features are extracted. For the 48 samples of each tissue, we simply count the number of samples in which the peptide is detected and divide by the total number of samples to get a fraction. The feature thus ranges from ca. 2% (1/48) to 100% (48/48).

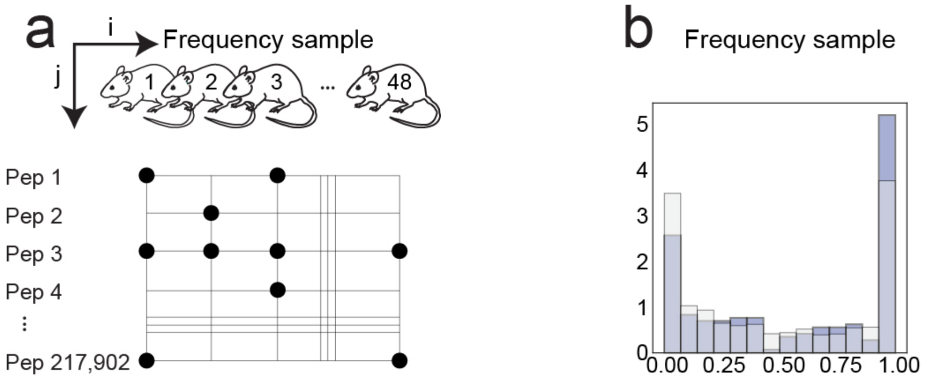

Supplementary Figure 22: a, illustration of how the features is derived. b, distribution of feature values for positive (blue) and negative (grey) training examples.

The formal method is thus:

$$x_{\text{Frequency sample},j} = \frac{1}{O}\sum_{k=1}^{k=O} \begin{cases} 0, & \text{if } I_{j,k} = 0 \\ 1, & \text{otherwise} \end{cases} \tag{3.12}$$

3.7 Frequency: PTMs

We searched the MS raw data for peptides with either N-terminal acetylation or C-terminal amidation. The same peptide sequence may therefore exist as 'unmodified', 'acetylated but not amidated', 'amidated but not acetylated', and as 'both acetylated and amidated'. Since these chemical modifications have been shown to be important for the stability and function of many known bioactive peptides, we wished to derive features which express the degree to which a given peptide is observed as being terminally modified. We did this for each observed peptide simply by computing the fraction of the abundance signal at its terminal position which originates from modified peptides. Supplementary Figure 23a shows a graphical illustration of the feature engineering with the distributions of the two resulting features in Supplementary Figure 23b and Supplementary Figure 23c.
The features extraction can be formally written as follows:

$$\begin{aligned} \text{let } ac_i &= \frac{H_{ac,i}}{H_i} \\ \text{let } am_i &= \frac{H_{am,i}}{H_i} \\ x_{\text{Frequency acetylation},j} &= ac_{p_{start(j)}} \\ x_{\text{Frequency amidation},j} &= am_{p_{stop(j)}} \end{aligned} \tag{3.13}$$

Using N-terminal amidation as example, the first step creates a vector (as long as the parent protein backbone) where each value is the fraction (of the abundance signal) coming from peptides ending

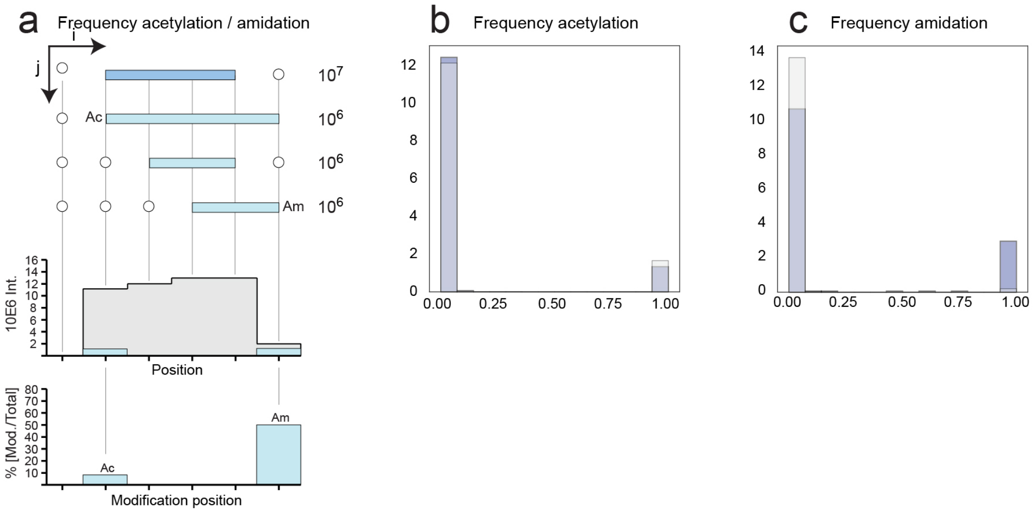

Supplementary Figure 23: a, illustration of how the features is derived. b, distribution of feature values for positive (blue) and negative (grey) training examples.

at that position with an amidation compared to the total signal from all observed peptides ending at the position. For amidated peptides, like GLP-1, this fraction was seen to be high.

3.8 Frequency Bond

This feature describes chemical bond preservation, as illustrated graphically in Supplementary Figure 24a.
This feature tries to find peptides where some of the bonds has been cut, It does that in 3 steps

- Create a Matrix J of all pairwise amino acid abundances
- Creates a vector B of all relative abundance between the pairs
- For each peptide it takes the sub vector of B_{sub} that corresponds to the peptide coordinates
- Finally we extract the worse preserved bond $min(B_{sub})$ and assigns it to the peptide

Mathematical what we do is: Pairwise calculate the abundance of all amino acids (J)

$$\text{let } J_i = [H_i, H_{i+1}] \tag{3.14}$$

Now we have a vector J the abundance of the N ($J_{i,0}$) and C ($J_{i,1}$) terminal amino acids, if their abundances are very different then this difference arise from a cleavage between the two amino acids Then we define B as the fraction of bonds shared by both amino acids: B is close to 1 when there are no cleavages and between 0 and 1 otherwise

$$\text{let } B_i = \frac{min(J_i)}{max(J_i)} \tag{3.15}$$

Then we take the sub vector of B that corresponds to the peptide coordinates (B_{sub}). Now we have a vector, but we would preferable a scalar feature, so we need to reduce this to a scalar, we could

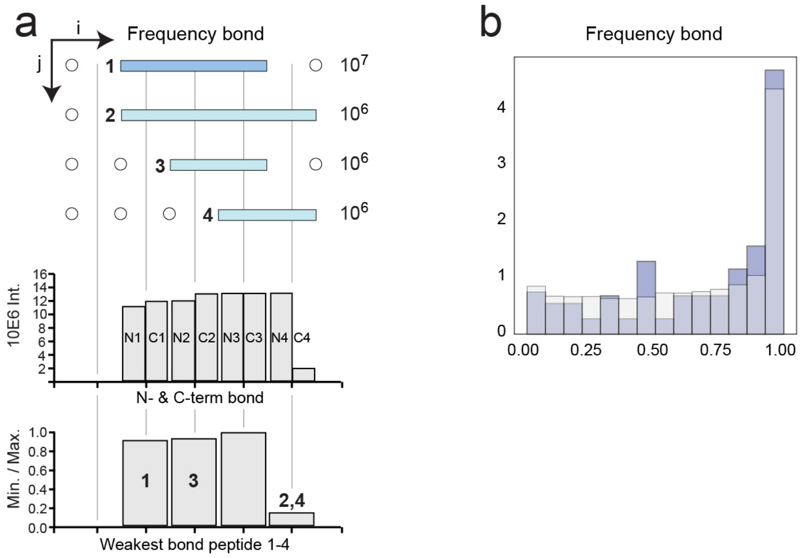

Supplementary Figure 24: a, illustration of how the features is derived. b, distribution of feature values for positive (blue) and negative (grey) training examples.

take the average or product of B_{sub} to get a feature corresponding to 'overall bond preservation' or 'unspecific cleavage', we however decided to take the lowest to get a feature expressing 'specific cleavage'.
the lowest of all this bond preservation is assigned to the peptide as a feature.

$$
\begin{aligned}
\text{let } B_{sub} = B_{p_{start}(j),p_{stop}(j)-1} \quad &= [B_{p_{start}(j)}, B_{p_{start}(j)+1}, \ldots, B_{p_{stop}(j)-1}] \\
x_{\text{Frequency bond},j} = min(B_{sub}) &
\end{aligned}
\tag{3.16}
$$

3.9 MS ladder

We noticed that some peptides seem to be digested from the terminals creating what looks like a stair case (ladder) of peptides when plotted. Thus we counted the number of alternative start and stop sites within a window size of $w = 10$ and calculated $ladder_{\text{stop}} = \frac{\text{starts here}}{\text{starts near}}$. We assumed that if a peptide was digested from either side it suggested unspecific degradation, and we therefore multiplied the N and C terminal fractions to create a single feature that is close to 0 when degraded and close to 1 otherwise.

$$
\begin{aligned}
&\text{let } w = 10 \\
ladder_{\text{start},i} &= \frac{C_{\text{start},i}}{\sum_{p_{start}(j)}^{p_{start}(j)+w} c_{\text{start},p_{start}(j)}} \\
ladder_{\text{stop},i} &= \frac{C_{\text{stop},i}}{\sum_{p_{stop}(j)-w}^{p_{stop}(j)} c_{\text{stop},p_{stop}(j)}} \\
x_{\text{MS Fraction ladder},j} &= ladder_{\text{start},p_{start}(j)} \times ladder_{\text{stop},p_{stop}(j)}
\end{aligned}
\tag{3.17}
$$

Conceptually there are many ways to try to extract this kind of feature, for instance, by varying the window size (w) and specifying the start/stop in normal or log space instead of as counts. Since the feature was a weaker predictor than expected, we did not pursue this to avoid too much tweeking and risk of over-fitting the data. We do, however, remain open to this being a potentially useful features if the degradation pattern can be expressed in a better way.

3.10 MS Intensity: penalty start/stop

We speculated that if a long peptide contains a short peptide with very high $x_{\text{MS Intensity start/stop}}$ then maybe this long peptide should be penalized to favor the shorter variant. To explore this concept, we constructed a penalty start/stop feature which summed all the start/stops within a peptide as follows:

$$x_{\text{Intensity penalty start},j} = \sum_{i=start_{p_{start}(j)}+1}^{stop_{stop}(j)-1} \Delta start_i$$
$$x_{\text{Intensity penalty stop},j} = \sum_{i=start_{p_{start}(j)}+1}^{stop_{stop}(j)-1} \Delta stopi \tag{3.18}$$

Where $\Delta start_i$ and $\Delta stop_i$ are defined in Equation3.8

$$x_{\text{MS Intensity cluster\_coverag},j} = \frac{\sum_{j=1}^{M} length_j}{N} \tag{3.19}$$

Where $length_j = p_{stop}(j) - p_{start}(j) + 1$

The feature did not turn out to be sufficiently predictive (see later).

3.11 MS Bool: observed

For the PPV assembly algorithm (See Chapter 5), we added an extra feature which simply expresses whether a given peptide was observed directly in the mass spectrometer or built in-silico via assembly of shorter overlapping fragments. This feature was set to 1 for the observed peptides and 0 for the in-silico assembled ones. For the in-silico assembled peptides, we furthermore set the feature $x_{\text{MS Frequency observed, j}}$ to 0.

The python code for computing all the features found in https://raw.githubusercontent.com/jancr/ppv/master/ppv/protein.py

Chapter 4

Machine Learning

4.1 Data Partitioning

Feature extraction provided us with 14 features which we used to distinguish the positive examples from the negatives, since there were only 207 positive examples. A classical 20% test 80% train split would only give us about 42 positive examples to evaluate our model, we therefore choose 5-fold nested cross validation to evaluate our models.

4.1.1 Nested Cross Validation

We split the data into 5 folds, for each fold we iterated the test set with the other 4 for folds used for cross validation. Thus we trained 20 models (5x4). A schematic Representation can be found in Supplementary Figure 25.
The data is partitioned into 5 folds, this is often implemented as two loops, the first loop picks one of the 5 folds as the test fold, the second loop picks one of the remanding 4 folds as the validation fold, Thus when the algorithm starts, fold 1 is used for testing, and fold 2 is used for validation, with fold 3-5 used for training. 4 iterations later we have 4 models, trained and validated on fold 2-5, each of which we can then test on fold 1, 16 iterations later, we have 5 groups of 4 models, which can be evaluated on their corresponding test set.

4.1.2 Stratified 5 Group Split

When splitting the data into five folds, then the primary concerns is feature leakage between the folds. This is usually solved by grouping peptides with correlated features into the same fold to prevent leakage, however when you group by one thing you risk your folds being stratifying by something that correlate with that one thing. In the present case some protein backbones have more amidations that other backbones, thus a grouped split will lead to a none random distribution of amidations across the folds. To counteract this we stratify by amidations, which tells the partitioning algorithm to do it's utmost to equalise the presence of amidations across the 5 folds.

Group by peptide clusters:
To solve the problem of leaking features from overlapping peptides, we created a Groups label where every "cluster of overlapping peptides" were grouped together into the same split, for example, if a protein backbone had the peptides 1-20, 15-25, 25-35, 36-45, and 36-50 then the peptides would be split into two clusters, one containing the first 3 (1-20, 15-25, and 25-35) and 1 containing the last 2 (36-45 and 36-50).

Stratify by amidation:
To create balanced folds, we split both on known yes/no and amidated yes/no, however, since you can only stratify by binary and categorical variables, for the purpose of stratifying the groups we dichotomized the continuous feature `MS Frequency amidation` to 1 if it was above 0.8 and 0 otherwise.
The grouping and stratification was done in sklearn using `sklearn.StratifiedGroupKFold`, where we stratified by positive and amidation and grouped by peptide cluster.

4.2 Model Specification

We Trained 5 classes of models (ordered by number of True Positives found in top 300):

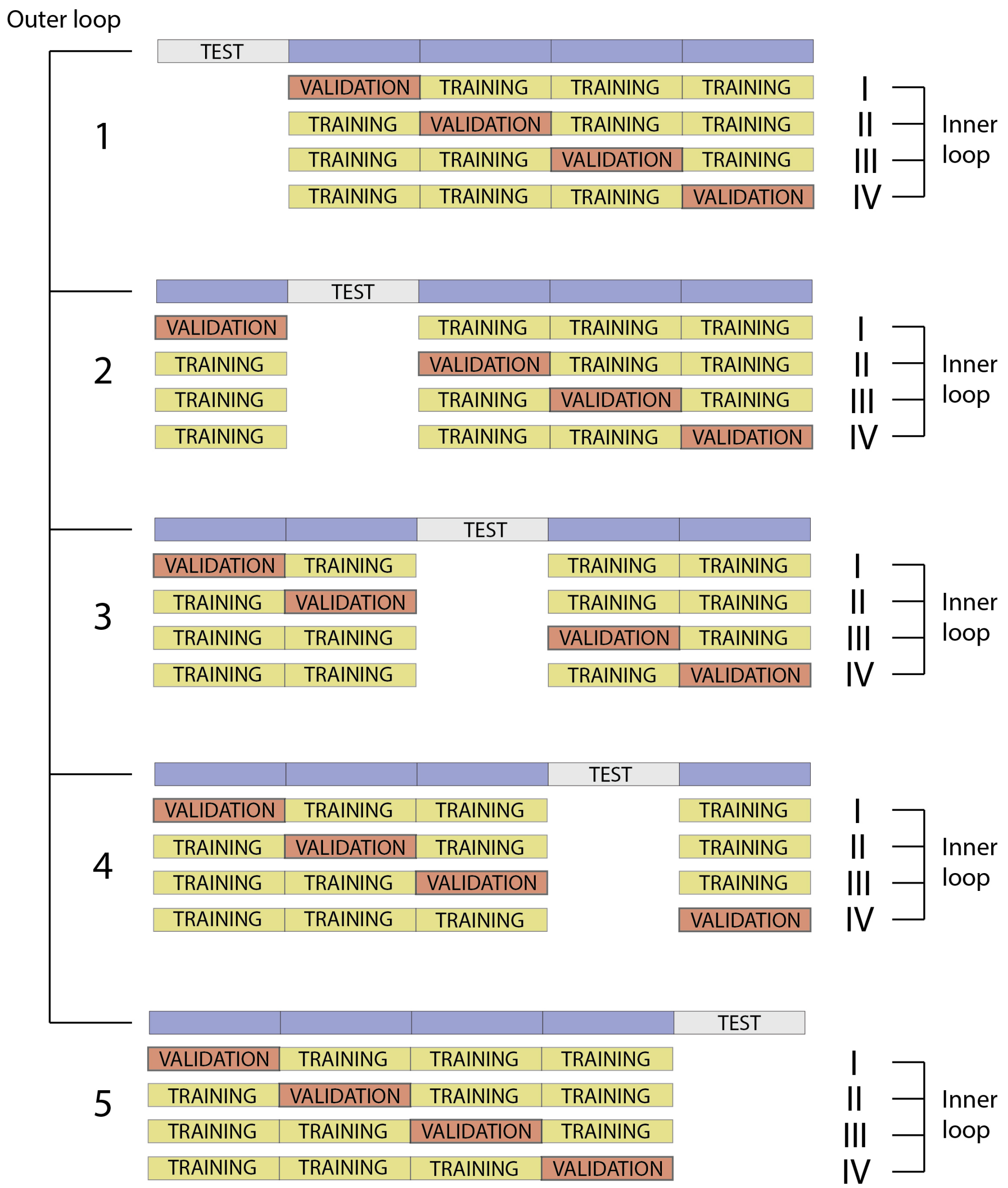

Supplementary Figure 25: Schematic representation of Nested Cross Validation, there are 5 outer loops which cycle trough which fold is used for testing, and 4 inner loop (per outer loop cycle) which cycle trough which fold is used for validation

1. PPV - logistic regression with L2 regularisation
2. Logistic regression with elastic net regularisation
3. Random forest
4. Logistic regression with L2 regularisation and training data up sampled by SMOTE[16], see Section 4.4.1 for more information.
5. Support Vector Machine (SVM) with a linear kernel (The dataset was to large to train a none linear kernel)
6. Null model, peptides sorted by MS abundance

For hyperparameter tuning we used the AUC on the validation set to determine the best hyperparameters.

4.3 Model Evaluation

The outcome of the previous sections was 5x20 Models with hyperparameters optimized on their validation folds.
The absolute and relative performance of the models was evaluated using ROC (or calibration) curves where the number of the known training peptides (our positive set) is plotted against the total number of peptides included above a given score cut-off. The allowed us to calculate the area under the curve (AUC).

The goal of our ML model was to train a classifier and use it on our data to find peptides that looked like the peptides annotated in Uniprot. Thus while we calculated AUC for all of our models, we were only interested in the highly predicted peptides. Therefore we plotted the Callibration curve but with a zoom on Top 300, See Supplementary Figure 26.
We also added a null model which was simply sorting the peptides according to their abundance, surprisingly that model performed far above random and got an AUC of 0.731, showing that known peptides tend to be more abundant that the other peptides we observe.
Overall, the logistic models (blue, red and purple) performed on par or slightly better than Random forest, which outperformed the SVM.
Amongst the 3 logistic models, the one using SMOTE performed much poorer on the early part of the curve. Elastic net performed slightly worse than pure L2 normalisation. Since elastic net pushes some features towards 0 the difference between the 20 elastic net models were larger than the 20 models regularized using L2, making the final model choice obvious, we picked logistic regression with L2 regularisation, as it had the highest number of TP on top 300, practically the same AUC as all the other top scoring models, and the added benefit of its predictions being less sensitive to the data partitioning.
We also compared to a previous model, called the Longest Peptide Variant (LPV) algorithm[13], which uses a predefined set of rules to assemble the longest possible peptides from observed peptide fragments (within certain restrictions). This model does not output a score or probability but simply creates a list of predicted, assembled peptides. It's performance is therefore visualized as a single point on the calibration curve (Supplementary Figure 26) and is seen to be on par with using the abundance (aka the **Null** model). We conclude that simply building and prioritizing the longest possible variant from overlapping fragments is not a good strategy.

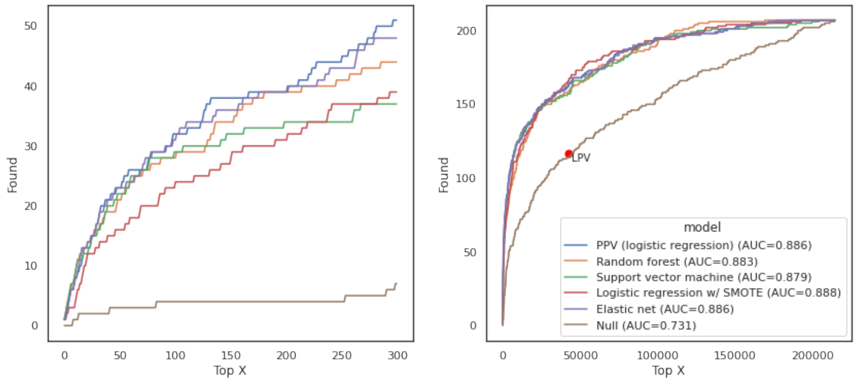

Supplementary Figure 26: Calibration curve over 6 models using different learning algorithms with a zoom onto the predictive performance among the top 300 highest scoring peptides. The dot illustrates the performance of the LPV algorithm[13].

4.3.1 Comparing to PeptideRanker

Our model only uses MS abundance-derived features, models using sequence derived-features also exist, such as PeptideRanker[1], Supplementary Figure 27 shows a comparison between out PPV algorithm and PeptideRanker on our data. While this plot suggests that our model is better than PeptideRanker, it's important to note that there are no overlapping features between the two models, as PeptideRanker uses only sequence features and PPV uses only MS features. Therefore they can be used together to find peptides that have strong MS and sequence-derived features.

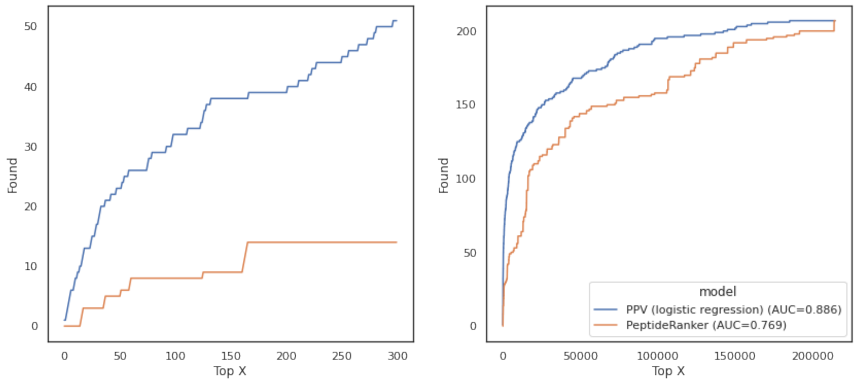

Supplementary Figure 27: Calibration curve of the PPV model and PeptideRanker.

[1]Note that PeptideRanker is trained on Uniprot and thus has all of our known peptides in its training set

4.3.2 Performance evaluation based on mislabeled peptides

As highlighted in the main paper, the model correctly predicts a number of peptides which were not included in our training set of annotated peptides and thus classified during training as negatives, but later identified as real peptides (see Supplementary Table 3). These serve as additional *in-silico* validation of the model predictions and show that the model is robust to misclassified training data. This is hardly surprising given the relatively modest number of input parameters in the model.

4.3.3 Enrichment of known cleavage sites and peptides from secreted proteins

Another way to evaluate the relevance of the predicted peptides is to look at other features (not used by the model) which are known to be characteristic of known peptides. Most - but not all - of the known bioactive peptides originate from proteins which are known to be secreted (most contain N-terminal signal peptides). Not counting the known peptides, about 18% of the peptides observed in our MS data are mapped to proteins which are known to be secreted. Among the top200 predicted peptides, this proportion is 28.5% showing that the PPV model enriches for peptides from secreted proteins among its novel predictions, even though the model has no knowledge of the secretion status. This strengthens our confidence in the biological relevance of the predictions.

Many of the known annotated peptides have been known for several decades, as have the details of their processing by proteases and other enzymes. Most well described are the prohormone convertases expressed in brain, pancreas and intestine (like PCKS1/PC1, PCSK2/PC2 and PCSK3/PC3) which cleave characteristic di-basic sequence motifs, like KR or GKR (where a glycine in position +1 can then be converted to form an amidated peptide).

To compare with the established knowledge, we constructed logo plots comparing the flanking regions of peptides predicted by the PPV model (the top500 peptides) to the rest of the peptides while excluding any known peptides from both groups (Supplementary Figure 28). The plots show a clear enrichment of certain sequence motifs in the N-terminal and C-terminal flanking regions of the predicted peptides. The motifs are rich in K and R which are known to be recognized by the prohormone convertases, as well as glycines (G) in position 1 which fits perfectly with amidation of the mature peptide. This is significant because we know that the signal is not coming from the already known bioactive peptides like glucagon, GLP-1, etc., since these were specifically removed from the analysis. The enrichment is seen for the novel predicted peptides which is a strong signal that our predictions are biologically meaningful, since the PPV model has no sequence derived knowledge including flanking regions. Tissues like brain, pancreas (and to a lesser extent) intestine (Ileum), and liver are dominated by the familiar di-basic motifs, whereas Muscle and the two fat tissues show enrichment of M, F and Y just upstream of the N-terminal, reflecting a different processing mechanism or degradation pattern in those tissues.

We performed the same analysis for the output of the simple rule-based LPV algorithm which simply outputs the longest peptides that can be assembled from shorter fragments. As can be seen (Supplementary Figure 28), the enrichment of motifs is much weaker which indicates that simply looking for the longest regions covered by peptide fragments is not an efficient strategy for identifying real bioactive peptides.

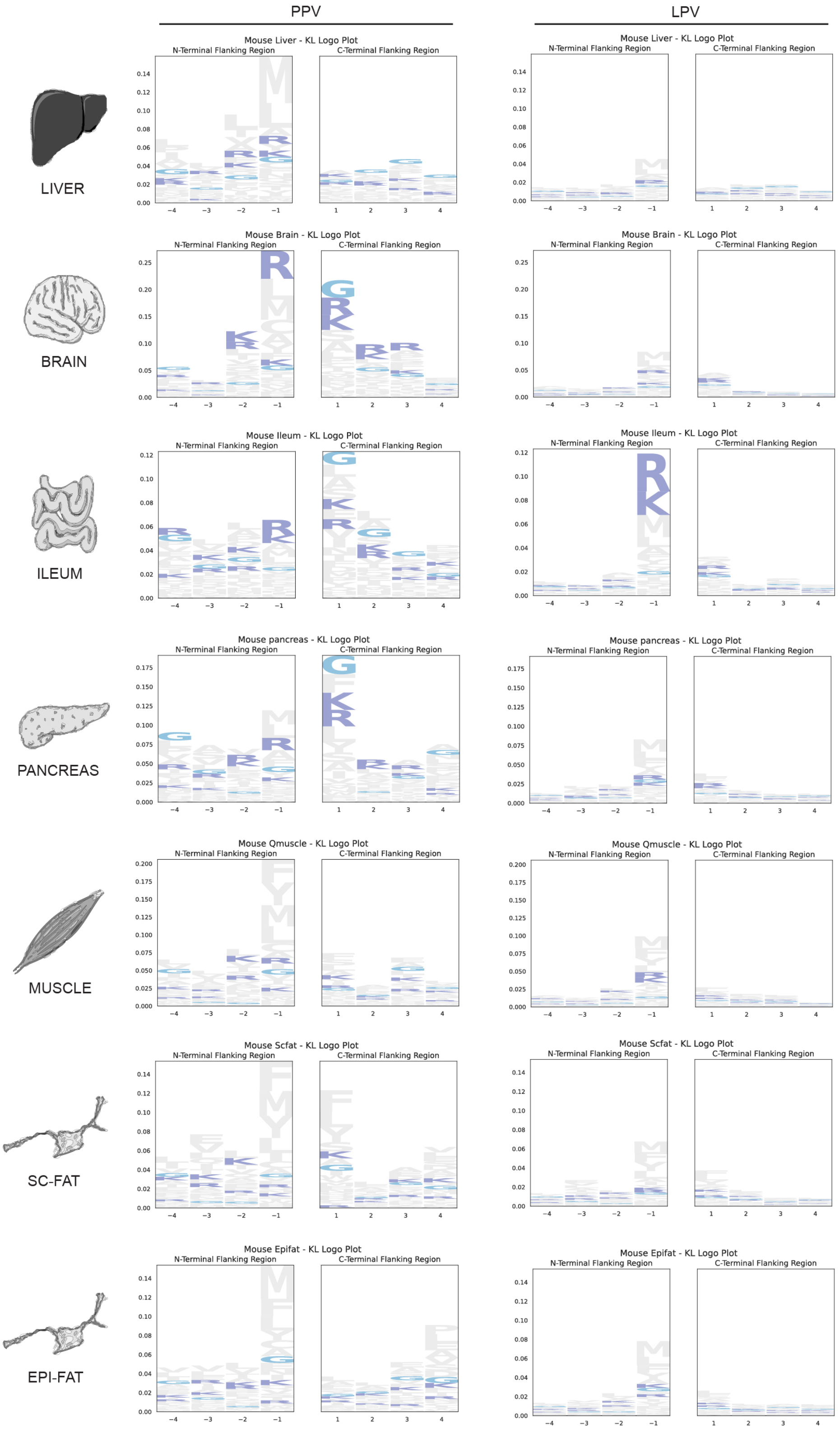

Supplementary Figure 28: Logo plots of N- and C-terminal flanking regions from LPV and PPV models. Longest-Peptide-Variant (LPV; right) and Predicted-Peptide-Variant (PPV; left) logo plots calculated by Kullback-Leibler divergence between all PPV/LPV peptides and all observed peptides which are not PPV/LPVs for each tissue. Lysine (K) and Argenine (R) are marked in dark blue, and Glycine (G) in light blue. LPV peptides are constructed using rules that builds exclusively the longest peptide variant within each cluster. For the PPV logo plots we used the top 500 highest scoring PPV peptides, and we removed all known peptides in both data sets.

4.4 Ablation Experiments

Note: When comparing the models we often use the logit scale, which is the logarithm of the odds ($logit(p) = ln(\frac{p}{1-p})$), We do this for two reasons 1. It is the natural scale of predictions from logistic models (which our final model happens to be), 2. It's a good way to "stretch" probabilities out, as an intuitive example lets use the odds 10:1, 1:1, 1:10, 1:100, 1:1000, 1:10000, when conveted to logits these are: 2.3, 0, -2.3, -4.6, -6.9, -9.2 when converted to probabilities they are 0.91, 0.5, 0.09, 0.01, 0.001, 0.0001, thus when plotting the probabilities, everything below 0.01 will be indistinguishable which is bad since most predictions are lower than 0.01, on the probability scale.

4.4.1 Oversampling of Positives

Because we have far fewer positive examples than negative examples we also tried to use SMOTE to upsample the positive training examples, this however did not improve performance. We suspect this is because there is a strong correlation between features. for example:

- if `MS Bool First`=1 then `MS Intensity` is almost always above 6, because the MS detection limit is about 10^6
- if `MS Bool First`=0, then `MS Intensity` is rarely above 2, because above 2 means that it's more than 100 times as abundant as the sum of all other overlapping peptides with longer N-terminals.

SMOTE will interpolate between the two archetypal peptides listed above, and create up sampled peptides where `MS Bool First`=0.5 and `MS Intensity Start`=4, as SMOTE ignores the conditional dependencies between features. Thus the synthetic peptide it creates looks very different from the rest of the data. Looking at Supplementary Figure 26 we can see that the AUC using SMOTE is about the same, but the number of TP in top 300 is 37 with SMOTE and 48 without.

4.4.2 Random undersampeling

Random forest and logistic regression are both models that are well equipped to handle data with a skewed class balance, especially the logistic model, as it has an intercept parameter that basically fits the class balance (in logit space), this means that if we under sample by a factor of e, then model average predicted probability will been shifted by 1 when transforming the probabilities to logits, thus under-sampling to 10% should change the odds by a factor of 10, and thus the log odds by $ln(10) = 2.3$ which is what we observe in Supplementary Figure 29:
A more intuitive way of quantifying this is looking at the overlap between the 300 highest scored peptides amongst the two models, which is 263 of 300. Because down sampling only shifts the predictions, we saw no reason to not train on all the data.

4.4.3 Removal of Long Peptides

The longest positive training example has a length of 42, whereas there are negatives of up to length 79. We did not a priori expect there to be any features that leaked length, but we did notice that longer peptides had slightly lower abundance. Furthermore if we keep the peptides longer than 42 then we risk biasing the model against whatever could correlated with length and thus under predict real peptides longer than 42. Thus we removed all peptides longer than length 42.
However, we want to use our model for inference on all peptides including the long ones. To get an understanding for the generalisability of our model, we trained a new model where we removed the

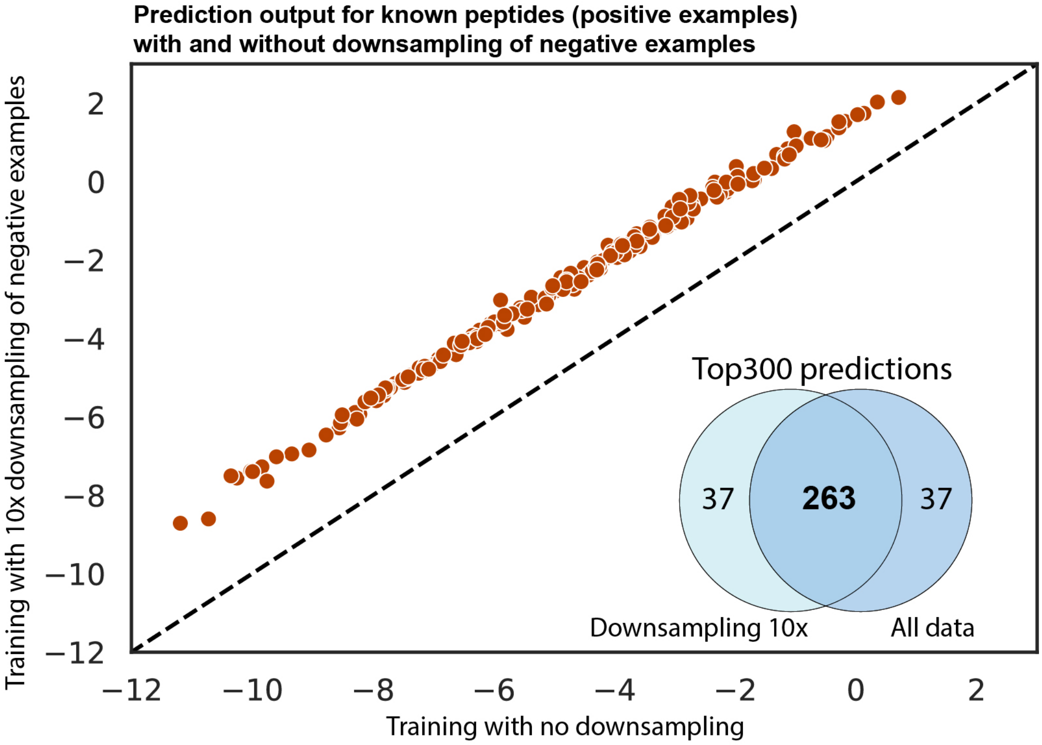

Supplementary Figure 29: Logit transformed predictions. The final Model (X) and the same model trained on data down-sampled to 10% (Y), Resulting in a 2.3 shift in the logits, Lower Right cornor show the overlap between the top 300 peptides amongst the two models

positive peptides of length 30-42 during training, the result of this can be seen in Supplementary Figure 30

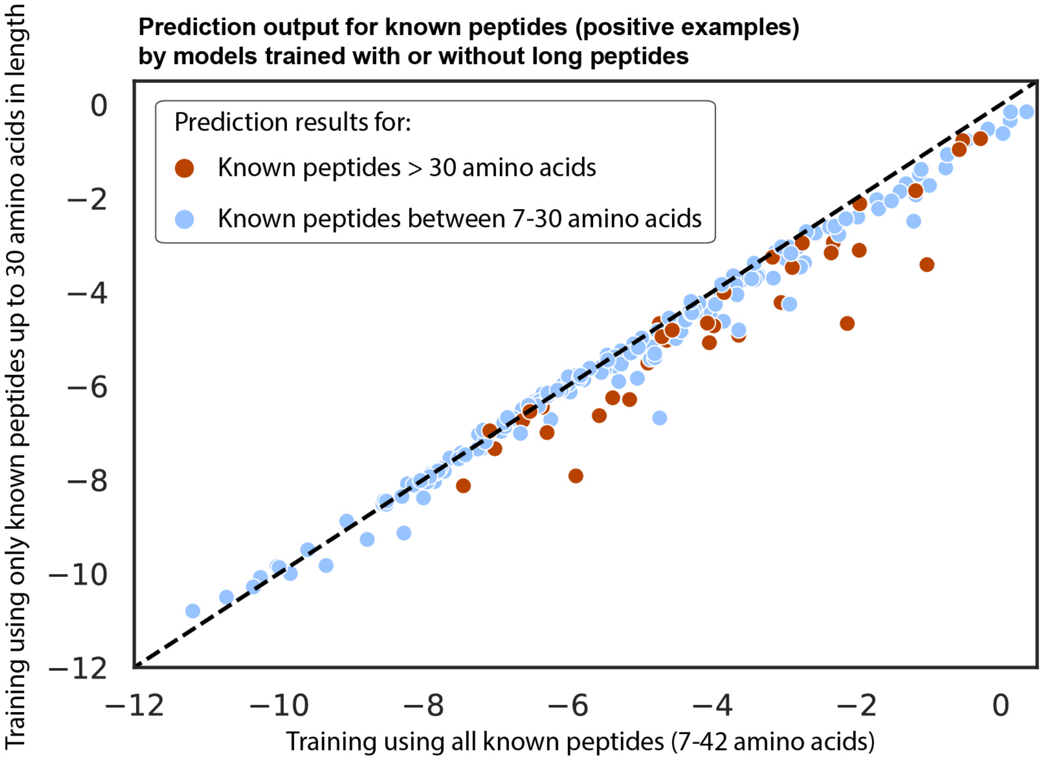

Supplementary Figure 30: 2 Models were trained, on on peptides of length 7-30 and another of length 7-42. X axis shows the 7-30 Model predictions in logits, Y axis shows the 7-42 Models predictions in logits. The known peptides were colored according to length.

Before looking at Supplementary Figure 30 let's think about what we expect from looking at Supplementary Figure 29:
When down-sampling the negative we expect virtually the same ordering as the uncertainly in model parameters comes mostly from the small number of positive training examples, now when downsampling the positive training examples instead (by removing peptides longer than 30), we expects the opposite, a small intercept shift and a larger shift in the other parameter resulting in a larger disagreement between the two models as compared to down-sampling the Negatives. Overall this is also what we see, there is still a strong correlation between the predictions, but the agreement is lower than in Supplementary Figure 29. Most data score slightly higher in the 7-42 Model, while generally the predictions from the two models are highly correlated suggesting that the 7-30 model can predict the 31-42 peptides, and by inferences that our 7-42 model can generalizes beyond peptides of length 42. It is worth noticing however that the long peptides tends to be scored relatively higher by the 7-42 model, suggesting that our model will be slightly under confident in long peptides, thus, if our final model scores a peptide longer than 42 high it could be considered extra strong evidence as it has a slight bias against that.

Chapter 5

The Assembly-PPV model

5.1 Motivation

As was evident from the sensitive analysis and visual inspection of the MS data (see main paper), some of the known uniprot peptides were not detected as perfect full length matches in our data but nonetheless manifested as clusters of proteolytically degraded fragments. In many cases, the borders of these fragment clusters fit perfectly with the full length sequence of the known peptides. To detect these, we would thus need to combine overlapping fragments into a predicted full length peptide.

To explore this approach, we applied an algorithm which assembles overlapping fragments into longer predicted peptides and were able to identify an additional 17 known peptides, including Amylin, Glicentin, Catestatin, Gastrin-releasing peptide, Somatostatin-28, PACAP-related-peptide and the CART peptide. A few were observed multiple times, giving us an additional 21 observations of known peptides.

The approach naturally also works for assembling novel/unknown bioactive peptides, as illustrated by the SCG1 peptide (385-435) which we identify and show in Figure 3c of the main paper. This novel peptide was observed as full length in pancreas and islet but in ileum and brain it could only by found by assembling shorter fragments.

5.2 Assembly of longer peptides

Imagine a simple hypothetical data set with only 4 peptides observed from a particular protein precursor:

- **Peptide A:** 20-30
- **Peptide B:** 22-32
- **Peptide C:** 25-32
- **Peptide D:** 50-60

Peptide A, B and C overlap with each other but not with Peptide D. One could hypothesize that a 5th peptide 20-32 exists, with peptide A and B simply being partially digested version of this longer variant. In contrast, there is no evidence to suggest that a longer variant of Peptide D should exists, since this peptide does not overlap with any other observed peptides.

To get all potential peptide combinations, we took all pairs of N- and C-terminal indexes from a peptide cluster in a protein backbone and created a sequence (Q) of all pairs where the N-terminal position comes before the C-terminal one:

$$\begin{aligned} Q &= \{[n_j, c_j] \mid n_j < c_j)\} \\ \text{where } n_j &= p_{start}(j) \\ \text{where } c_j &= p_{stop}(j) \end{aligned} \tag{5.1}$$

In the above example, this would create $Q = \{[20, 30], [20, 32], [22, 32], [25, 30], [25, 32]\}$ for the $\{A, B, C\}$ cluster. Applying this algorithm to the entire MS data set (on a per tissues basis), the number of peptide observations increases from 217,902 to 1,208,256, roughly corresponding to a 5x increase observations. Note, however, that the number of positive training examples (observations of known peptides) only increases from 207 to 228. So, the assembly algorithm increases the

amount of training data (mostly in the negative category) but at the same time introduces 5 times more data to predict on.

5.2.1 Model design and training

Using the expanded data set (assembled + observed peptides), we re-ran the PPV prediction model using the same 14 features identified above plus an additional boolean feature which simply encodes whether each peptide was observed or assembled (see Section 3.11). A new Test-Train-Validation split were performed on the upsampled dataset using the procedure outlined in Section 4.1, after which we again trained 20 Models using Nested Cross Validation.

5.2.2 Model parameters

A Boxplot depicting the parameters of the 20 PPV-assembly models can be found in Supplementary Figure 31.

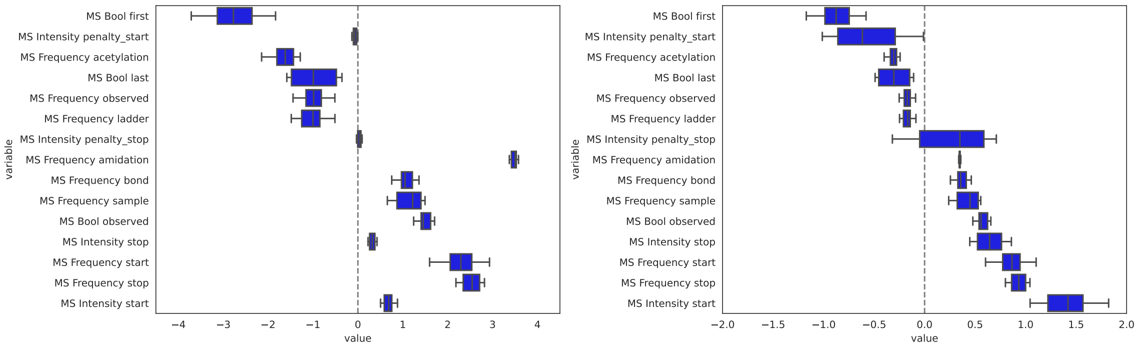

Supplementary Figure 31: Boxplot of the 15 Features used when training the PPV-Assembly model.

The directions of the coefficients (positive or negative weights) are quite comparable to those observed for the PPV, albeit with a tendency towards lower values and wider distributions. We interpret this as a sign that the patterns observed in the data are robust and extend also to the clusters from which the assembled peptides were built. Not surprisingly, the PPV-assembly model assigns a relatively large coefficient to the feature `MS Bool: Observed`, indicating that the model systematically shifts the likelihood of the assembled (non-observed) peptides downwards. This makes sense, considering the exhaustive nature of the assembly algorithm and in practice means that the signals in the other features need to be extra strong and convincing, when predicting on assembled peptides.

5.2.3 Model performance

The PPV-assembly model performs on par with the PVV model (with no assembly) among the top 300 predictions (Supplementary Figure 32). This again indicates that the training is robust to the noise which was potentially introduced by adding 5 times more assembled peptides.

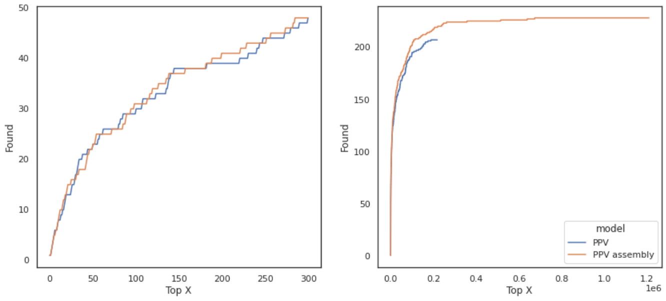

Supplementary Figure 32: Calibration curve of predictions from the PPV and PPV-assembly models. PPV model (blue) and PPV-assembly (orange). The assembly model artificially increase the data to 1.2 million peptides and slightly poorer performance among the top1000 highest scoring peptides (zoom).

5.3 Limitations and Future research

Although the PPV and PPV-assembly models are close to each other in performance, improvements may be achieved with a more sophisticated model for assembling peptides. That is, one which does not simply build all possible combinations.

We chose the logistic regression using nested cross validation for our PPV model due to its low risk of over-fitting the data and its high degree of explainability which allowed us to understand the impact individual features. As more annotations of known peptides become available in the future, it may be worth exploring more advanced, non-linear models and/or other features, either learned or human-engineered from the data.

Supplementary References

1. Park, C.B., Kim, M.S., Kim, S.C. A novel antimicrobial peptide from *Bufo bufo gargarizans*. *Biochem. Bioph. Res. Co*. 218, 408-413 (1996).

2. Ocaranza, M.P. *et al*. Counter-regulatory renin-angiotensin system in cardiovascular disease. *Nat. Rev. Cardiol*. 17, 116-129 (2020).

3. Kuoppala, A., Lindstedt, K.A., Saarinen, J., Kovanen, P.T., Kokkonen, J.O. Inactivation of bradykinin by angiotensin-converting enzyme and by carboxypeptidase N in human plasma. *Am. J. Physiol. Heart Circ. Physiol*. 278, H1069–H1074 (2000).

4. Yasothornsrikul, S*. et al*. Cathepsin L in secretory vesicles functions as a prohormone-processing enzyme for production of the enkephalin peptide neurotransmitter. *Proc. Natl. Acad. Sci. USA*. **100**, 9590–9595 (2003).

5. Cavasin, M.A., Rhaleb, N.E., Yang, X.P., Carretero, O.A. Prolyl oligopeptidase is involved in release of the antifibrotic peptide Ac-SDKP. *Hypertension*, **43**, 1140-1145 (2004).

6. Rioli, V. *et al*. Novel natural peptide substrates for endopeptidase 24.15, neurolysin, and angiotensin-converting enzyme. *J. Biol. Chem*. **278**, 8547–8855 (2003).

7. Zhao, Q., Piot, J.M. Neokyotorphin formation and quantitative evolution following human hemoglobin hydrolysis with cathepsin D. *Peptides*. **19**, 759–766 (1998).

8. Dagouassat, N., Garreau, I., Sannier, F., Zhao, Q., Piot, J.M. Generation of VV-hemorphin-7 from globin by peritoneal macrophages. *FEBS Lett*. **382**, 37–42 (1996).

9. Cunha, F.M. *et al*. Intracellular peptides as natural regulators of cell signaling. *J. Biol. Chem*. **283**, 24448-24459 (2008).

10. Diment, S., Martin, K.J., Stahl, P.D. Cleavage of Parathyroid Hormone in Macrophage Endosomes Illustrates a Novel Pathway for Intracellular Processing of Proteins. *J. Biol. Chem*. **264**, 13403-13406 (1989).

11. Devosse T. *et al*. Processing of HEBP1 by Cathepsin D Gives Rise to F2L, the Agonist of Formyl Peptide Receptor 3. *J. Immunol*. **187**, 1475-85 (2011).

12. Hamscher, G., Meyer, H.E., Feurle, G.E. Identification of proxenin as a precursor of the peptide xenin with sequence homology to yeast and mammalian coat protein alpha. *Peptides*. **17**, 889-893 (1996).

13. Secher, A. *et al*. Analytic framework for peptidomics applied to large-scale neuropeptide identification. *Nat. Commun*. **7**, 11436 (2016).

14. Brademan, D.R., Riley, N.M., Kwiecien, N.W., Coon, J.J. Interactive peptide spectral annotator: A versatile web-based tool for proteomics applications. *Mol. Cell Proteomics*. **18**, S193–S201 (2019).

15. Parker, B.L. *et al*. Multiplexed temporal quantification of the exercise-regulated plasma peptidome. *Mol. Cell. Proteomics*. **16**, 2055-2068 (2017).

16. Chawla, N.V., Bowyer, K.W., Hall, L.O., Kegelmeyer, W.P. SMOTE: Synthetic Minority Over-sampling Technique. *JAIR*. **16**. 321-357 (2002).
