# Supplementary material for: Combining mass spectrometry and machine learning to discover bioactive peptides
Source: Nat Commun. 2022 Oct 20;13:6235. doi: 10.1038/s41467-022-34031-z (PMC9584923; doi:10.1038/s41467-022-34031-z)
Supplement: Supplementary file 3 — Description of Additional Supplementary Files [file 41467_2022_34031_MOESM3_ESM.docx]

**Description of Additional Supplementary Files**

File Name: Supplementary Data 1

Description: Filtered and combined peptidomics output from Mascot and MaxQuant of n=48 mice. Peptide intensities reported as Log10.

File Name: Supplementary Data 2

Description: Proteomics output from n=3 brain samples of each strain. Protein intensities reported as LFQ intensities.

File Name: Supplementary Data 3

Description: Curated list of known and annotated peptides from Uniprot, SwePep, NeuroPep database. Peptides used for PPV training indicated.

File Name: Supplementary Data 4

Description: Peptidomics output from stimulated islets cells. Secretion output from n=4 independent replica consisting of n=500 cells.

File Name: Supplementary Data 5

Description: PPV predictions for all peptides with a score above 0.01. Each tissue is in one tab sheet, and one tab contains consolidated combined score for each peptide.

File Name: Supplementary Data 6

Description: PPV-assembly predictions for all peptides with a score above 0.01. Each tissue is in one tab sheet, and one tab contains consolidated combined score for each peptide.
