# Supplementary material for: Combining mass spectrometry and machine learning to discover bioactive peptides
Source: Nat Commun. 2022 Oct 20;13:6235. doi: 10.1038/s41467-022-34031-z (PMC9584923; doi:10.1038/s41467-022-34031-z)
Supplement: Supplementary file 10 — Reporting Summary [file 41467_2022_34031_MOESM10_ESM.pdf]

nature research

Corresponding author(s): Christian Toft Madsen

Last updated by author(s): Oct 4, 2022

Reporting Summary

Nature Research wishes to improve the reproducibility of the work that we publish. This form provides structure for consistency and transparency in reporting. For further information on Nature Research policies, see our Editorial Policies and the Editorial Policy Checklist.

Statistics

For all statistical analyses, confirm that the following items are present in the figure legend, table legend, main text, or Methods section.

n/a	Confirmed	
☐	☒	The exact sample size (n) for each experimental group/condition, given as a discrete number and unit of measurement
☐	☒	A statement on whether measurements were taken from distinct samples or whether the same sample was measured repeatedly
☐	☒	The statistical test(s) used AND whether they are one- or two-sided<br>*Only common tests should be described solely by name; describe more complex techniques in the Methods section.*
☒	☐	A description of all covariates tested
☐	☒	A description of any assumptions or corrections, such as tests of normality and adjustment for multiple comparisons
☐	☒	A full description of the statistical parameters including central tendency (e.g. means) or other basic estimates (e.g. regression coefficient) AND variation (e.g. standard deviation) or associated estimates of uncertainty (e.g. confidence intervals)
☐	☒	For null hypothesis testing, the test statistic (e.g. F, t, r) with confidence intervals, effect sizes, degrees of freedom and P value noted<br>*Give P values as exact values whenever suitable.*
☐	☒	For Bayesian analysis, information on the choice of priors and Markov chain Monte Carlo settings
☒	☐	For hierarchical and complex designs, identification of the appropriate level for tests and full reporting of outcomes
☐	☒	Estimates of effect sizes (e.g. Cohen's d, Pearson's r), indicating how they were calculated

Our web collection on statistics for biologists contains articles on many of the points above.

Software and code

Policy information about availability of computer code

Data collection	All mass spectrometry data was acquired on either a QExactive orbitrap or a QExactive HF orbitrap instrument (Thermo Scientific).
Data analysis	The peptidomics data used for encoding the PPV prediction algorithm was written in Scikit-learn (ver. 1.0.2) in python (ver. 3.9.7). PPV Feature frequency plots were made with Matplotlib (ver. 3.5.1). Calibration curves were made with Seaborn (ver. 0.11.2) in Jupyter (ver. 6.4.7). The PPV code is available in the GitHub repository: https://github.com/jancr/ppv, released under the MIT licence. The PPV code as also available from: DOI: 10.5281/zenodo.7140868

For manuscripts utilizing custom algorithms or software that are central to the research but not yet described in published literature, software must be made available to editors and reviewers. We strongly encourage code deposition in a community repository (e.g. GitHub). See the Nature Research guidelines for submitting code & software for further information.

Data

Policy information about availability of data

All manuscripts must include a data availability statement. This statement should provide the following information, where applicable:

- Accession codes, unique identifiers, or web links for publicly available datasets
- A list of figures that have associated raw data
- A description of any restrictions on data availability

MaxQuant software (ver. 1.5.6.2) was used to search proteomics brain data. Peptidomics data was searched with MaxQuant (ver. 1.6.0.1) and Mascot (ver. 2.6.2). Strain and tissue specificity plots were generated using UMAP library in Python (ver. 3.9.7). Data quality and assessment plots were made in R-studio (ver. 4.1.0; 2021-05-18), Seaborn (ver. 0.11.2), and Matplotlib (ver. 3.5.1) in Jupyter notebook (ver. 6.4.7). Gene ontology was done using geneontology.org. GraphPad Prism (ver. 7) was used for glucose uptake and GSIS data analysis. Source data supporting the findings in this study underlying dot plots and bar graphs are provided in the online source data file (Fig.3b, 4b-c, 5e, 7b, 8a-f, and the following supplementary figures; Supp. Fig 2a, 5b, 6b, 8a-c, 13a-d, 14a-d, 15a-d). Source data are provided

with this paper. The raw mass spectrometry data and processed search files are publicly available at the ProteomeXchange Consortium via the PRIDE partner repository with the data set identifier PXD022225 (https://www.ebi.ac.uk/pride/archive/projects/PXD022225/). Public peptide databases such as SwePep (http://www.swepep.org/), Uniprot (https://www.uniprot.org/) and NeuroPep (http://isyslab.info/NeuroPep/) were used for Supplementary Data 3.

Field-specific reporting

Please select the one below that is the best fit for your research. If you are not sure, read the appropriate sections before making your selection.

- [x] Life sciences
- [ ] Behavioural & social sciences
- [ ] Ecological, evolutionary & environmental sciences

For a reference copy of the document with all sections, see nature.com/documents/nr-reporting-summary-flat.pdf

Life sciences study design

All studies must disclose on these points even when the disclosure is negative.

Sample size	Peptidomics study involving n=12 mice in 4 different genetic or diet backgrounds (n=48 in total). This number is sufficient to detect differences in the peptidome as shown in the manuscript across genetic strain background. In training the PPV model it was desired to capture as much heterogeneity as possible in the peptidome from n=7 different tissues/organs, creating a total of n=336 samples analyzed. High-scoring PPV predictions were tested in n=3 diabetic mice for acute ability to influence blood glucose levels in-vivo, or in relevant in-vitro assays to minimize the number of experimental animals used. Any positive indication was reproduced in at least n=7 animals to increase statistical power. We chose n=3 mice to provide enough power for observing a minimum 20% change in BG, and n=7~10 was chosen based on a minimum of 10% change in BG based on historical variation within internal studies in this experimental paradigm.
Data exclusions	No animals were excluded from the acquisition of the mass spectrometry data, downstream analysis or PPV training, except one rawfile (Diabetic mouse_09 from Sc. Fat) which was truncated during MS acquisition and subsequently removed from downstream analysis. Peptides below 7 amino acids in length or with a Mascot score below 20 were discarded computationally. For hydrodynamic gene delivery animals were excluded if they exhibited poor status after tail veil injection, and not used for terminal plasma collection.
Replication	Peptides were screened in-vivo in n=3 db/db mice, and replicated in n=7 or n=10 animals. All in-vitro data was at least replicated as two independent biological replica each with two technical replica in total.
Randomization	Mass spectrometry data acquisition was randomized for strain background within each tissue to minimize strain batch effects. For blood glucose measurements mice older than 11 weeks and with blood glucose levels higher than 16 mM were selected and allocated to different treatment groups by randomization based on blood glucose levels. Animals were assigned to groups, based on BG so that all groups had equivalent and representative mean and SD blood glucose not significantly different from one another.
Blinding	Mass spectrometry data acquisition was not blinded for the strain identity. For training the logistic (PPV) and comparative non-linear models we used nested 5-fold cross validation to ensure that reported performance metrics are based only on data unseen by the models during training and optimization. The regression coefficients are reported from 20 models. Blood glucose measurements were recorded by a researcher not familiar with the grouping and without a record of the group identities.

Reporting for specific materials, systems and methods

We require information from authors about some types of materials, experimental systems and methods used in many studies. Here, indicate whether each material, system or method listed is relevant to your study. If you are not sure if a list item applies to your research, read the appropriate section before selecting a response.

Materials & experimental systems

n/a	Involved in the study	
[ ]	[x]	Antibodies
[ ]	[x]	Eukaryotic cell lines
[x]	[ ]	Palaeontology and archaeology
[ ]	[x]	Animals and other organisms
[x]	[ ]	Human research participants
[x]	[ ]	Clinical data
[x]	[ ]	Dual use research of concern

Methods

n/a	Involved in the study	
[x]	[ ]	ChIP-seq
[x]	[ ]	Flow cytometry
[x]	[ ]	MRI-based neuroimaging

Antibodies

Antibodies used	Monoclonal mouse HUI018 was made in Novo Nordisk against human insulin using classical hybridoma technology. The polyclonal antibody pAB 4077 fractions E+F was raised against rat insulin 1+2 in guinea pigs and isolated by chromatography. Antibodies are described in Andersen et al. 1993 (https://pubmed.ncbi.nlm.nih.gov/8472350/).
Validation	Plasma insulin level was measured by an in house developed Luminescence Oxygen Channeling Immunoassay (LOCI). 5 ug/mL, 35 uL/well of mAb HUI018 conjugated acceptor beads is used for plate coating, followed by incubation with detection antibody at 6 ug/mL,

10 uL/well of Biotin-4077 E+F. Qualification in LOCI is done using spike recovery and dilution recovery test, and the assay has been extensively described elsewhere; Poulsen F. et al. 2007 (https://pubmed.ncbi.nlm.nih.gov/17259593/), Rojas JM. et al. 2018 (https://pubmed.ncbi.nlm.nih.gov/30343647/).

Eukaryotic cell lines

Policy information about cell lines

Cell line source(s)	Primary hepatocyte cells were isolated from male Sprague Dawley Crl:CD(SD) rats (Charles River). 3T3-L1 MBX fibroblast cells were purchased from ATCC, #CRL3242. The INS1E clone-21 was generated in-house by stable transfecting a proinsulin-luciferase reporter construct (licensed from the Broad Institute). Rat insulinoma-derived INS1 cell line was originally developed in the lab of Claes Wollheim (https://pubmed.ncbi.nlm.nih.gov/14592952/).
Authentication	Primary hepatocytes are used immediately as detailed in methods. 3T3-L1 MBX is authenticated from the supplier by comparing the short-tandem repeat profile with the ATCC Human Cell STR Database. INS1E was not authenticated but reported to have a stable differentiated phenotype over 116 passages representing 2.2-yr (https://pubmed.ncbi.nlm.nih.gov/14592952/).
Mycoplasma contamination	Test for mycoplasma contamination was negative.
Commonly misidentified lines (See ICLAC register)	No commonly misidentified cell lines were used.

Animals and other organisms

Policy information about studies involving animals; ARRIVE guidelines recommended for reporting animal research

Laboratory animals	Animals from Taconic (lean C57bl/6J mice), Jackson Laboratory (diet induced obese C57bl/6J mice fed 60% high fat diet (HF) and C57bl/6J mice fed 10% low fat diet (LF)) or Charles River (C57bl/KS db/db) and C57bl/KS db/+). Mice were all male and 12 weeks of age upon arrival, and tissue collected at 15 weeks (WT,DB) and at age 26 weeks (LF,HF). Male diabetic BKS.Cg-Dock7m +/+ Leprdb/J (stock no: 000642) strain introduced from the Jackson Laboratory USA and used for blood glucose measurements at age 11 weeks. Male Sprague-Dawley rats Crl:CD(SD) rats (Charles River) were used for primary hepatocyte isolation at age 8 weeks.
Wild animals	This study did not involve wild animals.
Field-collected samples	This study did not involve field-collected samples.
Ethics oversight	Animal studies were carried out in accordance with the Danish Act on Experiments on Animals - Appendix A of ETS 123 and EU Directive 2010/63. Animal protocols was approved by the Institutional Animal Care and Use Committee of Novo Nordisk Research Center China and Ethical Review Council in Novo Nordisk A/S (Permission no. 2015-15-0201-00616).

Note that full information on the approval of the study protocol must also be provided in the manuscript.
